# Direct transfer of zinc between plants is channelled by common mycorrhizal network of arbuscular mycorrhizal fungi and evidenced by changes in expression of zinc transporter genes in fungus and plant

**DOI:** 10.1111/1462-2920.15542

**Published:** 2021-05-10

**Authors:** Alessio Cardini, Elisa Pellegrino, Stéphane Declerck, Maryline Calonne‐Salmon, Barbara Mazzolai, Laura Ercoli

**Affiliations:** ^1^ Institute of Life Sciences, Sant'Anna School of Advanced Studies Piazza Martiri della Libertà 33 Pisa 56127 Italy; ^2^ Université catholique de Louvain, Earth and Life Institute, Applied Microbiology, Mycology Croix du Sud 2, Box L7.05.06 Louvain‐la‐Neuve 1348 Belgium; ^3^ Center for Micro‐BioRobotics, Istituto Italiano di Tecnologia Viale Rinaldo Piaggio 34, 56025 Pontedera Pisa Italy

## Abstract

The role that common mycorrhizal networks (CMNs) play in plant‐to‐plant transfer of zinc (Zn) has not yet been investigated, despite the proved functions of arbuscular mycorrhizal fungi (AMF) in crop Zn acquisition. Here, two autotrophic *Medicago truncatula* plants were linked by a CMN formed by *Rhizophagus irregularis*. Plants were grown *in vitro* in physically separated compartments (Donor‐C and Receiver‐C) and their connection ensured only by CMN. A symbiosis‐defective mutant of *M. truncatula* was used as control in Receiver‐C. Plants in both compartments were grown on Zn‐free medium, and only the leaves of the donor plants were Zn fertilized. A direct transfer of Zn was demonstrated from donor leaves to receiver shoots mediated by CMN. Direct transfer of Zn was supported by changes in the expression of fungal genes, *RiZRT1* and *RiZnT1*, and plant gene *MtZIP2* in roots and *MtNAS1* in roots and shoots of the receiver plants. Moreover, Zn transfer was supported by the change in expression of *MtZIP14* gene in AM fungal colonized roots. This work is the first evidence of a direct Zn transfer from a donor to a receiver plant via CMN, and of a triggering of transcriptional regulation of fungal‐plant genes involved in Zn transport‐related processes.

## Introduction

In the recent decade, an increasing number of studies have reported the ability of arbuscular mycorrhizal fungi (AMF) to connect plants belonging to the same or different species, genera and families in common mycorrhizal networks (CMNs). The capacity of AMF to develop such CMNs is related to their wide host range, even though a variability in host specificity among AMF has been found (Öpik *et al*., 2009; Veresoglou and Rillig, 2014; Ciccolini *et al*., 2016; Davison *et al*., 2020). These CMNs have attracted the attention of the scientific community due to their presumed roles in the transfer of nutrients and photosynthetic C between interconnected plants (e.g., Newman *et al*., 1994; He *et al*., 2003; Simard *et al*., 2003; Selosse *et al*., 2006; Voets *et al*., 2008; Fellbaum *et al*., 2014), thus playing key roles in the functioning of ecosystems (van der Heijden *et al*., 1998).

CMNs are complex structures whose density varies with AM fungal isolate and interconnected plant species (Giovannetti *et al*., 2004; Avio *et al*., 2006). The high frequency of anastomoses (hyphal fusions; Kirk *et al*., 2008; 44%–63%) between extraradical hyphae of the same AM fungal isolate, spreading from the roots of different plants, sustains the establishment and functionality of CMNs (Giovannetti *et al*., 2004; Avio *et al*., 2006; Croll *et al*., 2009). Connection between plants can also be established from a mycelium growing from an AM fungal colonized plant to a not inoculated one as reported by Voets and colleagues (2008, 2009) in a bi‐compartmented *in vitro* cultivation system. In field conditions, a single individual mycelium belonging to a cluster within the *Glomus* clade was found to link the roots of *Hieracium pilosella*, covering up to 10 m in length (Rosendahl and Stukenbrock, 2004). Further laboratory studies suggested that ubiquitous AM fungal species, such as *Funnneliformis mosseae* and *Rhizophagus intraradices*, interconnected tomato (*Solanum lycopersicum*) and faba bean (*Vicia faba*) plants separated by a distance up to 15 cm (Song *et al*., 2010; Babikova *et al*., 2013).

Several studies have postulated the transport of nutrients (i.e. phosphorus, P, nitrogen, N and carbon, C) from plant to plant via CMNs in pot and field conditions (e.g., Simard and Durall, 2004; Jakobsen and Hammer, 2015; Wipf *et al*., 2019). For instance, P absorption by young maize plants was greater in unploughed than in ploughed soil, and this was also confirmed in pots with plants grown in soil cores collected from unploughed and ploughed plots (Read and Birch, 1988). One plausible explanation is that the established AM fungal network provides the way for P inflow to the young plants. In native grasslands, about 20% of the foliar ^32^P applied to *Plantago erecta* was transferred to the shoots of interconnected plants (Chiariello *et al*., 1982). In a microcosm, P concentration in seedlings of *Bromus erectum* and *Brachipodium pinnatum* was increased when plants were linked by a CMN (van der Heijden, 2004). Moreover, in a pot experiment with plants interconnected by a CMN, P was preferentially transported to established cucumber (*Cucumis sativus*) as compared with tomato (*Solanum lycopersicum*) seedlings (Merrild *et al*., 2013). Nitrogen transfer was also reported between two interconnected plants of *Plantago lanceolata* grown in pot conditions (Eissenstat, 1990). In pot studies, it was also shown that inorganic forms of N were transferred from N_2_‐fixing plants to non‐fixing plants interconnected by AM fungi (e.g., van Kessel *et al*., 1985; Bethlenfalvay *et al*., 1991; Frey and Schüepp, 1993; Martin *et al*., 1995; Johansen and Jensen, 1996). However, although in all cited experiments the transfer of nutrients was proven to occur between plants linked by AMF, it was not possible to firmly discriminate the direct transfer channelled by CMN from the one mediated by, e.g., soil diffusion and uptake by hyphae or roots. Thus, to better understand the importance of CMN in the transfer of nutrients, it is important to exclude or at least to minimize as much as possible indirect transfer caused by diffusion, by transport by unwanted microorganisms or by hyphae release and uptake by roots.

In the last decades, *in vitro* cultivation systems associating AMF with root organs (e.g., carrots – see review by Fortin *et al*., 2002) or whole plants (Lalaymia and Declerck, 2020) have been used to investigate transport [e.g., P, N, Caesium (Cs)] from a root‐free compartment to a root compartment or between CMN‐connected plants growing in physically separated compartments. Both systems are adequate to minimize indirect transfer of elements because of the absence of unwanted contaminants and the strict separation of the receiver roots or whole plant from the element to study. For instance, Nielsen and colleagues (2002) using root organ cultures (ROC) of carrot were able to demonstrate the uptake of P by the extraradical mycelium developing in a root‐free hyphal compartment (HC), its translocation to a root compartment (RC) and further transfer within the roots. The same *in vitro* system was used to investigate the transfer of C and N from HC to RC, but both elements remained in the colonized roots of the receiver plant (Pfeffer *et al*., 2004; Toussaint *et al*., 2004; Jin *et al*., 2005) due to the absence of a true sink, the shoot. Therefore, a system on whole plants was developed by Voets and colleagues (2005) to study the transport of elements from HC to roots and shoots in the RC. This system was used by Dupré de Boulois and colleagues (2006) to investigate the transport of P and Cs from HC to roots and shoots in the RC. For P, ~21% of the initial ^33^P supplied to the HC was transferred to the shoots, while for Cs, only 1.8% of the initial ^134^Cs supplied in the HCs was found in the shoots of *Medicago truncatula*, suggesting the major role of AMF in supplying P to plants, while for Cs, colonized roots were the preferred sink. The system of Voets and colleagues (2005) was further extended to two plants interconnected by a CMN (Voets *et al*., 2008) for studying plant‐to‐plant transfer of C (Voets *et al*., 2008) and Cs (Gyuricza *et al*., 2010). If C was transferred into the receiver roots, it was not transferred from roots to shoot, while for Cs, similar observations as in the study of Dupré de Boulois and colleagues (2006) were made with little accumulation of Cs in shoots. Importantly, in the study of Voets and colleagues (2008) an extremely low amount of C was detected in the non‐colonized control plants suggesting that a tiny amount of C transported by hyphae from RC to HC was released into the medium and further taken up by the non‐AM‐colonized plant. The values were, however, significantly lower than in the AM‐colonized plants suggesting an effective direct transfer between interconnected plants. More recently, the system of Voets and colleagues (2008) was extended to demonstrate the change of expression of defence genes in healthy plants connected to diseased plants (Alaux *et al*., 2020). Overall, these studies open huge avenues to unravel the full range of functions a web plays in plant‐to‐plant interactions and (agro)‐ecosystems functioning.

The role played by CMNs in interplant transfer of micronutrients, such as copper (Cu), iron (Fe) and zinc (Zn), has not been investigated yet, despite the demonstrated positive roles of AMF in micronutrient plant acquisition (Lehmann *et al*., 2014; Lehmann, 2015; Ercoli *et al*., 2017; Coccina *et al*., 2019; Pellegrino *et al*., 2020). Among micronutrients, Zn is essential for growth and health of plants and is involved in various physiological functions (Vallee and Auld, 1990; Sasaki *et al*., 1998; Broadley *et al*., 2007). Zinc deficiencies reduce plant growth and quality of food products, whereas Zn excess can be toxic in food and reduce plant growth. Thus, plants have developed complex homeostatic mechanisms that ensure optimal cellular Zn concentration when grown in soil with limiting or toxic levels of Zn (Grusak *et al*., 1999; Sinclair and Krämer, 2012). The active transport of Zn in plants has been largely studied and many membrane transporters belonging to several families have been identified (e.g., Sinclair and Krämer, 2012; Olsen and Palmgren, 2014). One of the most studied families involved in the transport of Zn into the cytoplasm is the Zinc‐Iron‐Regulated Transporter (*ZRT‐IRT*), called *ZIP*. The *ZIP1*, a vacuolar transporter, was found to be upregulated in roots and leaves of *M. truncatula* deficient in Zn (López‐Millán *et al*., 2004; Durmaz *et al*., 2011; Milner *et al*., 2013). The *ZIP2*, a plasma membrane transporter into the stele promoting Zn accumulation in the xylem parenchyma, was found to be upregulated in *M. truncatula* roots and stems, especially at high Zn concentrations (Burleigh *et al*., 2003). Moreover, other studies found that Zn chelators, such as the non‐proteinogenic amino acid nicotianamine (NA), enabled the intercellular and phloem mobility of Zn in plant (Curie *et al*., 2008; Deinlein *et al*., 2012). Recently, other ZIP genes, such as *ZIP6*, *ZIP7* and *ZIP14*, were reported to be upregulated when plants of *M. truncatula* were inoculated with AMF under a gradient of Zn concentration in soil (Watts‐Williams *et al*., 2017, 2020). Specifically, *ZIP6* was upregulated under low and medium concentration of Zn in soil (Watts‐Williams *et al*., 2017), whereas *ZIP7* was highly upregulated irrespective to Zn concentration in soil (Watts‐Williams *et al*., 2020). Finally, *ZIP14* was exclusively expressed in AMF‐colonized plants and upregulated irrespective to Zn concentration in soil. MtZIP14 was localized in AMF colonized root cells and specifically in the peri‐arbuscular membrane (PAM). By contrast, the mechanism of Zn homeostasis in AMF was less investigated and only one Zn transporter, belonging to the Cation Diffusion Facilitator (CDF) family, the *GintZnT1*, was identified in *Rhizophagus irregularis* (González‐Guerrero *et al*., 2005, 2016; Tamayo *et al*., 2014). The *GintZnT1* is likely participating in vacuolar sequestration, since it can transport Zn^2+^ out of the cytosol (González‐Guerrero *et al*., 2005, 2009). Tamayo and colleagues (2014) identified by an *in silico* study other putative Zn transporters in *R. irregularis*.

**Fig. 1:**
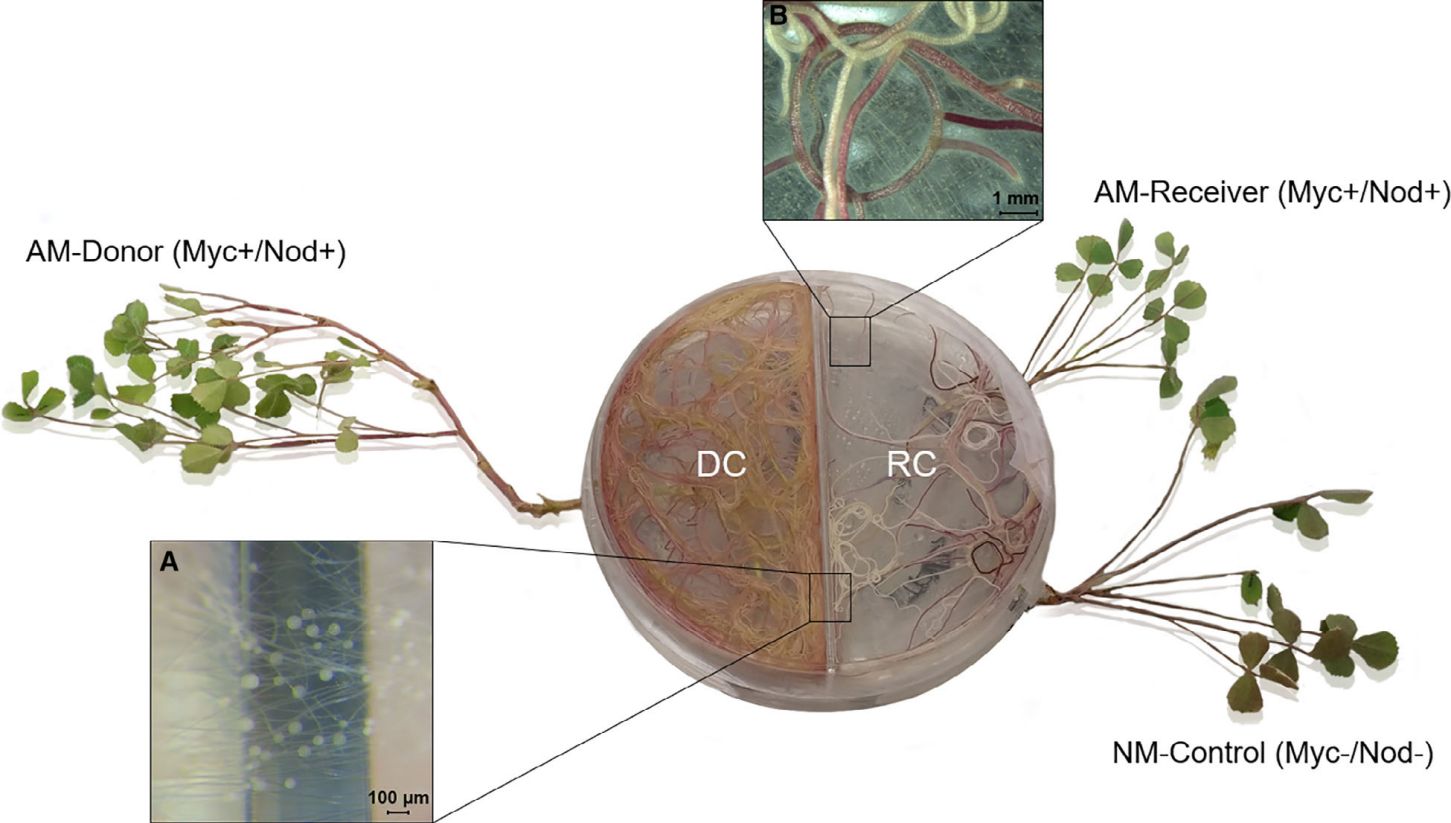
Bi‐compartmented autotrophic *in vitro* culture system with an arbuscular mycorrhizal (AM) donor plant (AM‐Donor), an AM receiver plant (AM‐Receiver) and non‐mycorrhizal mutant plant (NM‐Control) of *Medicago truncatula* (Voets *et al*., 2008). The genotype of the AM‐Donor and AM‐Receiver plants was the wild type J5 and the one of the NM‐Control plants was its isogenic mycorrhiza defective mutant TRV25. A five‐day old AM‐Donor seedling was inoculated with the AM fungus *Rhizophagus irregularis* (MUCL 41833) 2 weeks after the insertion into the donor compartment (DC). Eight weeks after inoculation, the AM‐Receiver and the NM‐Control seedlings were inserted in the receiver compartment (Receiver‐C). In the Figure, details of the common mycorrhizal network are shown: AM fungal hyphae crossing the partitioning wall (A) and extraradical AM fungal mycelium with roots of the AM‐Receiver plants (B)

In the present study, a CMN formed by the AM fungus *R. irregularis* MUCL 41833 was established *in vitro* between two *M. truncatula* plants, one donor plant (AM‐Donor, hereafter) fertilized with Zn on the leaves and one receiver plant (AM‐Receiver, hereafter). Both plants were grown on a Zn‐free medium in a bi‐compartmented system that physically separated the donor compartment (Donor‐C) from the receiver compartment (Receiver‐C; Fig. 1). Zinc availability in soil can be low and is always patchy, and under agricultural systems the homogeneity of application of foliar fertilizers can be variable, from low to medium, according to the application techniques (Ciccolini *et al*., 2017). Thus, the process of Zn transfer by CMN is ecologically important as it may improve the redistribution of Zn in agro‐ecosystems as well as in natural ecosystems. Therefore, we investigated whether the transfer of Zn was directly associated to the connecting hyphae and postulated that if the fungus transfer Zn from a leaf fertilized plant to an AM‐Receiver plant, a higher Zn concentration and variation in the expression of plant Zn‐transporter genes is observed compared with a non‐mycorrhizal control plant (NM‐Control; Hypothesis 1). The NM‐Control is a mycorrhizal defective mutant isogenic line of *M. truncatula* that was added to the Receiver‐C to ascertain that transfer was essentially attributable to direct mechanisms. Moreover, to minimize the role of indirect Zn transfer, Zn concentration in the media of Donor‐C and Receiver‐C was analysed. In addition, we hypothesized that after Zn application a larger part of Zn remains in the AM‐Donor, whereas the remaining part is transferred to the AM‐Receiver (Hypothesis 2). This will result in a significant higher Zn concentration and variation in plant gene expression profile in the AM‐Donor as compared with the AM‐Receiver. Moreover, to verify that Zn transfer by CMN does not occur in absence of Zn application, we set up the same *in vitro* system (Fig. 1), excluding Zn treatment to leaves of the AM‐Donor. In this no‐Zn system, we expected no difference in plant Zn concentration and gene expression profile neither between AM‐Receiver and NM‐Control or between AM‐Donor and AM‐Receiver (Hypothesis 3). Finally, to further support the evidence of Zn transfer by CMN from AM‐Donor to AM‐Receiver, comparisons of Zn‐fertilized and unfertilized plants were performed on plant Zn concentration and gene expression separately for AM‐Donor, AM‐Receiver and NM‐Control, whereas the same comparison was performed on fungal Zn‐transporter genes separately for AM‐Donor and AM‐Receiver.

In addition, we evaluated the adequacy of the CMN *in vitro* cultivation system for the plant‐to‐plant Zn transfer study, verifying plant and fungus growth under Zn and no‐Zn application. We expected that, according to differences in plant age (due to the sequence of the operations of the experimental set‐up), the AM‐Donor was larger than the AM‐Receiver, and that the AM‐Receiver grew similarly to the NM‐Control. Moreover, on the basis of literature data on the AM fungus growth in *in vitro* system, we expected that in an adequate system the fungus extensively grew (e.g., larger mycelium) and produced more spores in Receiver‐C than in the Donor‐C. Finally, to further support the adequacy of the system excluding any toxic effect of Zn, comparisons of plant growth traits under +Zn and −Zn treatments were performed separately for AM‐Donor, AM‐Receiver and NM‐Control, and comparisons of fungal growth traits were performed separately for Donor‐C and Receiver‐C.

## Results

### Adequacy of the CMN in vitro cultivation system for plant‐to‐plant Zn transfer study

Shoot and root dry weights (SDW and RDW) of AM‐Donor were threefold to fourfold higher as compared with AM‐Receiver in −Zn treatment as well as +Zn treatment (Table S1). By contrast, SDW and RDW of AM‐Receiver and NM‐Control were similar under both +Zn and –Zn treatments. Similarly, stem length and number of leaves were significantly higher in AM‐Donor as compared with AM‐Receiver under both +Zn and −Zn treatments (on average +102% and +157% respectively), and did not vary between AM‐Receiver and NM‐Control. Root length of AM‐Donor was six‐fold higher as compared with AM‐Receiver under both +Zn and −Zn treatments (Table 1). By contrast, root length of AM‐Receiver was similar to NM‐Control under both +Zn and −Zn treatments.

**Table 1:** Root length, arbuscular mycorrhizal (AM) fungal root colonization and length of colonized roots of wild type *Medicago truncatula* donor (AM‐Donor), receiver (AM‐Receiver) and AM‐defective mutant control (NM‐Control) plants, 5 days after application of zinc (0.1 mg plant^−1^; +Zn) on the leaves of the AM‐Donor plants or in absence of Zn (−Zn). The AM‐Donor and AM‐Receiver plants were linked by the extraradical mycelium of the AM fungus *Rhizophagus irregularis* (MUCL 41833).

Plant^a^	Root length	AM fungal colonization	AM fungal colonized root length
	cm	%	cm
AM‐Donor +Zn	719.4 ± 52.6	52.4 ± 2.1	372.7 ± 19.1
AM‐Receiver +Zn	130.3 ± 19.3	46.4 ± 1.8	59.3 ± 6.6
NM‐Control +Zn	104.1 ± 18.6	0.0 ± 0.0	0.0 ± 0.0
AM‐Donor −Zn	720.7 ± 33.6	45.6 ± 3.0	325.9 ± 15.9
AM‐Receiver −Zn	110.7 ± 22.5	48.0 ± 2.5	54.2 ± 12.9
NM‐Control −Zn	106.0 ± 17.9	0.0 ± 0.0	0.0 ± 0.0
*Treatments compared (P‐values of linear orthogonal contrasts)*			
AM‐Donor versus AM‐Receiver +Zn	**<0.001^b^**	**0.020**	**<0.001**
AM‐Receiver versus NM‐Control +Zn	0.597	**<0.001**	**0.004**
AM‐Donor versus AM‐Receiver −Zn	**0.001**	0.243	**<0.001**
AM‐Receiver versus NM‐Control −Zn	0.706	**0.001**	**0.007**

^a^ AM‐Donor: *Medicago truncatula* Gaertn., cv. ‘Jemalong’ wild type (line J5; Myc+/Nod+); AM‐Receiver: *M. truncatula* Gaertn., cv. ‘Jemalong’ wild type (line J5; Myc+/Nod+); NM‐Control: *M. truncatula* Gaertn., cv. ‘Jemalong’ mutant line TRV 25 (Myc−/Nod−). One‐way analysis of variance (ANOVA) was performed to test the effect of plant type and orthogonal contrasts were used to discriminate the differences between AM‐Donor and AM‐Receiver plants and between AM‐Receiver and NM‐Control plants. Values are means ± standard error of five replicates.

^b^ In bold statistically significant values at *P* ≤ 0.05.

Under Zn application, AM fungal root colonization percentage was significantly higher in AM‐Donor as compared with AM‐Receiver. By contrast, under −Zn treatment, AM fungal root colonization was similar in AM‐Donor and AM‐Receiver. Moreover, under +Zn and −Zn treatments, the AM fungal colonized root length was on average sixfold higher in AM‐Donor as compared with AM‐Receiver. As expected, NM‐Control (i.e., the mycorrhizal defective mutant isogenic line TRV25) did not show any trace of mycorrhizal colonization. The number of spores was significantly higher in the Receiver‐C as compared with the Donor‐C under both +Zn and −Zn treatments (Table 2). Similarly, hyphal length per root length and hyphal length per AM fungal colonized root length were significantly higher in the Receiver‐C as compared with the Donor‐C irrespectively of Zn application. By contrast, total hyphal length and hyphal density were similar in the Donor‐C and the Receiver‐C under both Zn treatments.

**Table 2:** Hyphal length, number of spores, hyphal length per root length, hyphal length per arbuscular mycorrhizal (AM) fungal root length and hyphal density in the donor and receiver compartments (Donor‐C and Receiver‐C respectively), 5 days after application of zinc (0.1 mg plant^−1^; +Zn) on the leaves of the AM‐Donor plants or in absence of Zn (−Zn). The AM‐Donor and AM‐Receiver plants were linked by the extraradical mycelium of the AM fungus *Rhizophagus irregularis* (MUCL 41833).

Compartment^a^	Hyphal length	*N*° spores	Hyphal length per root length	Hyphal length per AM fungal root length	Hyphal density
	cm	*N*	cm cm^−1^	cm cm^−1^	cm cm^−3^
Donor‐C +Zn	1560 ± 102	3462 ± 466 a^b^	2.19 ± 0.18 a	4.18 ± 0.22 a	98.1 ± 6.4
Receiver‐C +Zn	2047 ± 267	9532 ± 1068 b	17.8 ± 4.3 b	37.5 ± 7.9 b	128.7 ± 16.8
Donor‐C −Zn	1621 ± 297	3897 ± 504 a	2.3 ± 0.5 a	5.0 ± 1.0 a	102.0 ± 18.7
Receiver‐C −Zn	2287 ± 373	8048 ± 1144 b	22.7 ± 3.8 b	47.8 ± 8.5 b	143.8 ± 23.4

^a^ In Donor‐C the *Medicago truncatula* Gaertn., cv. ‘Jemalong’ wild type (line J5; Myc+/Nod+) was grown. In Receiver‐C the *M. truncatula* Gaertn. cv. ‘Jemalong’ wild type (line J5; Myc+/Nod+) and the mutant *M. truncatula* Gaertn., cv. ‘Jemalong’ mutant line TRV 25 (Myc−/Nod−), were grown. For each Zn treatment, *t*‐test was performed to test the effect of the compartment on the AM fungal traits. Values are means ± standard error of five replicates.

^b^ Values followed by different letters are significantly different at *P ≤* 0.05, according the post‐hoc Tuckey‐B test.

Overall, the application of Zn did not modify the phenotype of the plants as evidenced by *t*‐test conducted on all plant traits for each plant type (AM‐Donor, AM‐Receiver and NM‐Control (Table S2). Similarly, the application of Zn did not impact fungal parameters as evidenced by *t*‐test (Tables S2 and S3). Finally, the number of active hyphae crossing the partition wall separating Donor‐C from Receiver‐C did not significantly differ between +Zn and −Zn treatments (306.6 ± 42.2 and 244.4 ± 38.4 respectively).

### Shoot and root Zn concentrations in plants linked by CMN

**Fig. 2:**
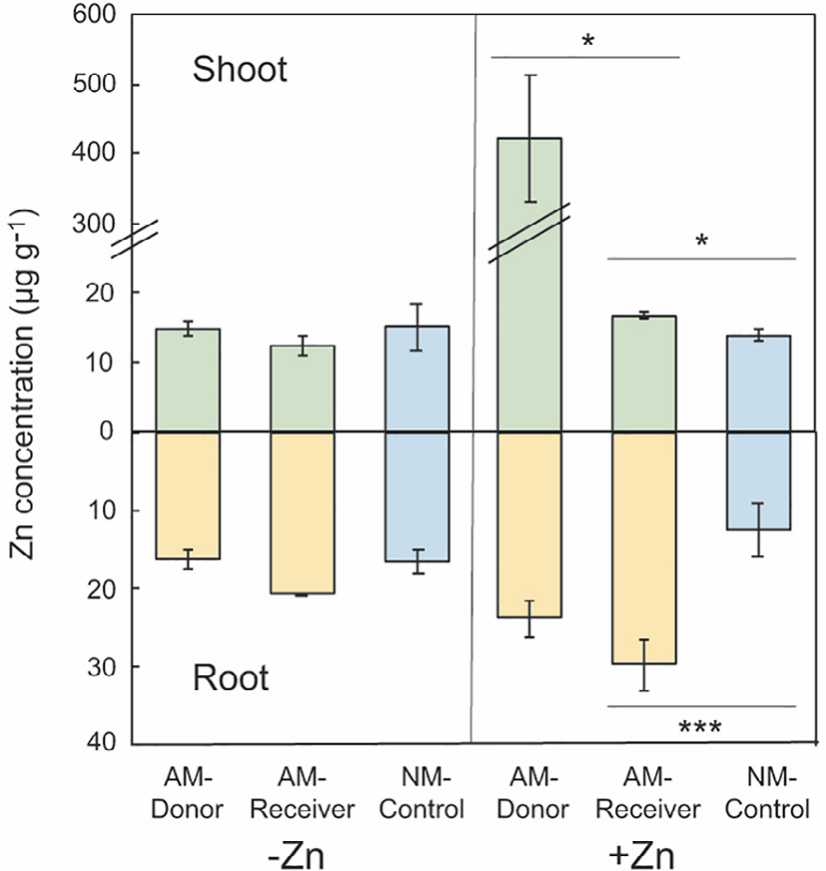
Zinc concentrations (μg g^−1^) in shoots and roots of 16‐weeks‐old wild type *Medicago truncatula* donor plants (AM‐Donor; Myc+/Nod+), six‐weeks‐old wild type receiver *M. truncatula* plants (AM‐Receiver; Myc+/Nod+) and its isogenic mycorrhiza defective mutant control plants (NM‐Control; Myc‐/Nod‐), 5 days after zinc (Zn) application of 0 and 0.1 mg plant^−1^ (−Zn and + Zn respectively) on the leaves of the AM‐Donor plants. The AM‐Donor and AM‐Receiver plants were linked by the extraradical mycelium of the arbuscular mycorrhizal (AM) fungus *Rhizophagus irregularis* (MUCL 41833). Values are means ± standard error of three replicates (*n* = 3). One‐way analysis of variance (ANOVA) was performed to test the effect of plant type and orthogonal contrasts were used to discriminate the differences between AM‐Donor and AM‐Receiver plants and between AM‐Receiver and NM‐Control plants. Different symbols indicate significant differences between plants (*: 0.01 < *P* ≤ 0.05; **: 0.001 ≤ *P* ≤ 0.01; ***: *P* < 0.001).

Shoot Zn concentration of AM‐Receiver was significantly higher (by 21%) than that of NM‐Control (Fig. 2; Table S4). Moreover, shoot Zn concentration of AM‐Donor was 25‐fold higher as compared with the AM‐Receiver under +Zn treatment. By contrast, under −Zn treatment, no significant differences were noticed in the shoot Zn concentration of AM‐Donor, AM‐Receiver and NM‐Control.

Under Zn application, root Zn concentration of AM‐Receiver was significantly higher (139%) than that of NM‐Control, while root Zn concentration of AM‐Donor was similar to that of AM‐Receiver (on average 26.8 μg g^−1^; Fig. 2; Table S4). By contrast, under −Zn treatment no significant differences were noticed among AM‐Donor, AM‐Receiver and NM‐Control.

Shoot Zn concentration was about 28‐fold higher in AM‐Donor in the +Zn treatment as compared with the AM‐Donor in the −Zn treatment, while root Zn concentration was 47% higher (Table S2). Shoot Zn concentration was 35% higher in AM‐Receiver in the +Zn treatment as compared with AM‐Receiver in the −Zn treatment, while root Zn concentration was about 44% higher. By contrast, Zn concentration of shoot and root of NM‐Control was not significantly modified by Zn treatment.

### Plant and fungal gene expressions in plants linked by CMN

**Fig. 3:**
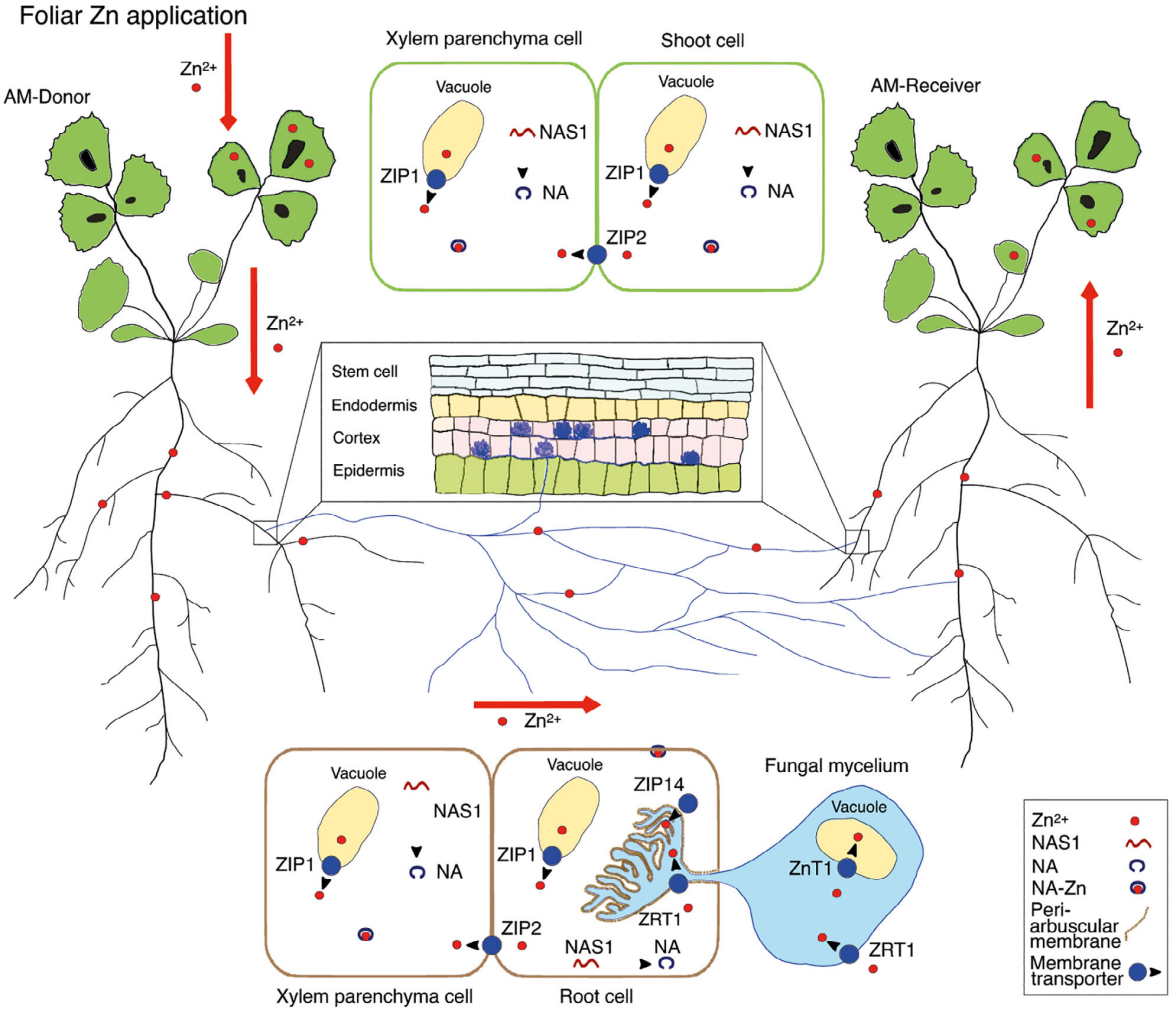
Schematic representation of the studied Zn transport‐related proteins for uptake, sequestration, and redistribution of Zn after foliar application to the arbuscular mycorrhizal (AM) *Medicago truncatula* donor plant (AM‐Donor; Myc+/Nod+) linked by the common mycorrhizal network to the *M. trucatula* AM‐Receiver plant (Myc+/Nod+). A putative transport protein (ZIP1) involved in the vacuolar transport of Zn into the cytoplasm (López‐Millán *et al*., 2004), a putative transport protein (ZIP2) involved in the cellular Zn influx into the xylem parenchyma cells (Burleigh *et al*., 2003) and an enzyme synthesizing nicotianamine (NAS1; Deinlein *et al*., 2012) are indicated in the scheme within the cells of shoots and roots. The nicotianamine (NA) and the ligand complex Zn‐nicotianamine (Zn‐NA) are also indicated. The RiZnT1 protein likely participating in Zn vacuolar sequestration (González‐Guerrero *et al*., 2005), the putative transporter RiZRT1 likely participating in the influx of Zn through the fungal plasma membrane (Tamayo *et al*., 2014) and the putative Zn transporter ZIP14 localized in AMF colonized root cells and specifically in the peri‐arbuscular membrane (Watts‐Williams *et al*., 2020) are indicated in the scheme within the cells of the colonized roots.

**Fig. 4:**
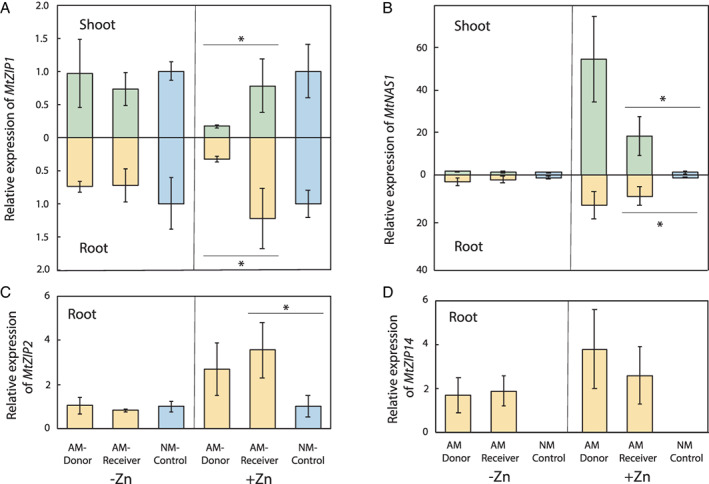
Relative expression of the Zinc‐Iron‐Regulated Transporter ZIP genes, *MtZIP1* in shoots and roots (A), *MtZIP2* and *MtZIP14* in roots (C,D), and of the nicotianamine synthase gene (*MtNAS1*) (B) of 16‐weeks‐old wild type *Medicago truncatula* donor plants (AM‐Donor; Myc+/Nod+), six‐weeks‐old wild type receiver *M. truncatula* plants (AM‐Receiver; Myc+/Nod+) and its isogenic mycorrhiza defective mutant control plants (NM‐Control; Myc−/Nod−), 5 days after zinc (Zn) application of 0 and 0.1 mg plant^−1^ (−Zn and +Zn respectively) on the leaves of the AM‐Donor plants. The AM‐Donor and AM‐Receiver plants were linked by the extraradical mycelium of the arbuscular mycorrhizal (AM) fungus *Rhizophagus irregularis* (MUCL 41833). The relative expression analysis of the genes was done by the double standardization method that requires the reference genes and the control treatment (Livak and Schmittgen, 2001; Vandesompele *et al*., 2002). The transcript levels of actin‐101 (*MtACT‐101*) and elongation factor 1‐α (*MtEF1‐α*) were used as reference (Nicot *et al*., 2005) and the transcript level of the NM‐Control plants was used as control. Values are means ± standard error of five replicates (*n* = 5). One‐way analysis of variance was performed to test the effect of plant type and orthogonal contrasts were used to discriminate the differences between AM‐Donor and AM‐Receiver plants and between AM‐Receiver and NM‐Control plants. Different symbols indicate significant differences between plants (*: 0.01 < *P* ≤ 0.05; **: 0.001 ≤ *P* ≤ 0.01; ***: *P* < 0.001).

A schematic representation of the Zn transport‐related proteins for uptake, sequestration, and redistribution of Zn after foliar application to the AM‐Donor linked by the CMN to the AM‐Receiver is reported in Fig. 3. Under Zn application, the gene *MtZIP1* was significantly downregulated in AM‐Donor shoots and roots (−78% and −73% respectively) as compared with the AM‐Receiver, which showed expression patterns similar to NM‐Control (Fig. 4A; Table S5). The gene *MtZIP2* was not expressed in the shoots of the plants of *M. truncatula* under both +Zn and −Zn treatments (Table S5). Nevertheless, when Zn was applied to the leaves of AM‐Donor, the gene *MtZIP2* was similarly expressed in the roots of AM‐Donor and AM‐Receiver, and *MtZIP2* gene expression was about threefold higher in AM‐Receiver than NM‐Control (Fig. 4C; Table S5). Moreover, the gene *MtNAS1* was significantly upregulated in the shoots of AM‐Donor (about threefolds) as compared with the AM‐Receiver that also showed a significant upregulation (about 18 folds) as compared with NM‐Control (Fig. 4B; Table S5). However, in roots, the gene *MtNAS1* was similarly upregulated in AM‐Donor and AM‐Receiver and *MtNAS1* gene expression was about ninefold higher in AM‐Receiver than in NM‐Control. By contrast, under −Zn treatment, the expression of *MtZIP1*, *MtZIP2* and *MtNAS1* in shoots and roots did not vary between AM‐Donor and AM‐Receiver and neither between AM‐Receivers and NM‐Control (Fig. 4; Table S5).

Under Zn foliar application, the AM‐responsive *MtZIP14* gene, whose protein was specifically localized in the peri‐arbuscular membrane, was similarly expressed in the AM‐Donor and AM‐Receiver, whereas it was not expressed in NM‐Control (Fig. 4D; Table S5). Moreover, under −Zn treatment, *MtZIP14* was upregulated in both AM‐Donor and AM‐Receiver.

**Fig. 5:**
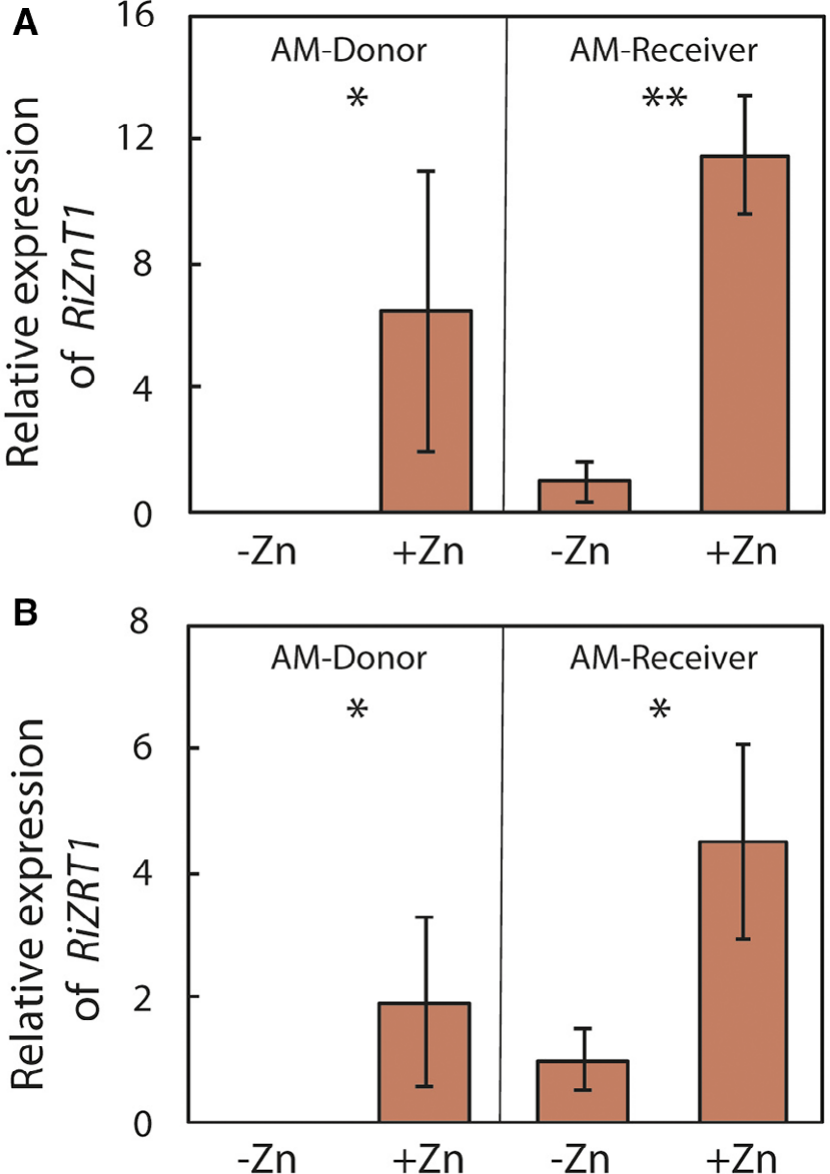
Relative expression of the *Rhizophagus irregularis RiZnT1* (A) and *RiZRT1* (B) genes in roots of 16‐weeks‐old wild type *Medicago truncatula* donor plants (AM‐Donor; Myc+/Nod+), and 6‐weeks‐old wild type receiver *M. truncatula* plants (AM‐Receiver; Myc+/Nod+), 5 days after zinc (Zn) application of 0 and 0.1 mg plant^−1^ (−Zn and +Zn respectively) on the leaves of the AM‐Donor plants. The AM‐Donor and AM‐Receiver plants were linked by the extraradical mycelium of the arbuscular mycorrhizal (AM) fungus *Rhizophagus irregularis* (MUCL 41833). The relative expression analysis of the genes was done by the double standardization method that requires the reference genes and the control (−Zn) (Livak and Schmittgen, 2001; Vandesompele *et al*., 2002). The transcript level of the 28S ribosomal subunit (*Ri28S*) was used as reference (Nicot *et al*., 2005) and the transcript level of the AM‐Receiver roots in the −Zn treatment was used as control. Values are means ± standard error of five replicates (*n* = 5). Pairwise comparisons of Zn‐fertilized and unfertilized plants were performed by *t*‐test separately for AM‐Donor and AM‐receiver plants. Different symbols indicate significant differences between plants (*: 0.01 < *P* ≤ 0.05; **: 0.001 ≤ *P* ≤ 0.01; ***: *P* < 0.001).

Under Zn application, the genes *RiZnT1* and *RiZRT1* were both expressed in the roots of AM‐Donor and AM‐Receiver (Fig. 5; Table S5). As expected, under Zn application, *RiZnT1* and *RiZRT1* genes were not expressed in NM‐Control. Conversely, under −Zn treatment, the genes *RiZnT1* and *RiZRT1* were not expressed in AM‐Donor, very little expressed in AM‐Receiver and not expressed in NM‐Control (Fig. 5; Table S5). The transcript levels of the *Ri28S* were similar among the roots of AM‐Donor and AM‐Receiver in the +Zn and −Zn treatments (Fig. S1).

The evidence of Zn transfer from donor to AMF‐receiver plants was further supported by the differences in plant gene expression between +Zn and −Zn treatments in AM‐Donor and AMF‐Receiver (Table S6). Zinc transfer was additionally supported by the differences on fungal gene expression between +Zn and −Zn treatments in AM‐Donor versus AMF‐Receiver.

## Discussion

It is only in the recent years that the role of CMNs in the transfer of macronutrients (e.g., N and P) between plants has been shown with AMF (e.g., He *et al*., 2003; Mikkelsen *et al*., 2008), while it was not studied for micronutrients, such as Zn. Controversy about whether transfer is direct through CMNs or partly indirect through soil may arise in experiments conducted under pot conditions (He *et al*., 2003; Bücking *et al*., 2016). Indeed, the direct contribution of connecting hyphae can only be ascertained in a context where indirect effects, attributed to diffusion in soil, hyphal exudation and decay, impact of microbial communities or biofilm formations, can be excluded. In the present study, a partition wall, physically separating the two compartments of the Petri plate, allowed excluding the diffusion pathway of Zn from the Donor‐C to the Receiver‐C and subsequent uptake by the roots of the receiver plants. Moreover, the introduction of the NM‐Control plant in the Receiver‐C allowed a precise control of the Zn released into the growth medium due to hyphal exudation and decay. The transfer of Zn from AM‐Donor to AM‐receiver plants via the CMN was supported by the significant increase in Zn concentration of the shoots of the AM‐Receiver plants and by the changes in the expression of the fungal genes *RiZRT1* and *RiZnT1*, as well as of *MtZIP2, MtZIP14* and *MtNAS1* in the roots and of *MtNAS1* in the shoots of *M. truncatula*. However, only the use of radioactive Zn isotopes as tracers would have provided conclusive evidence of the direct Zn transfer between plants connected by CMN (Zhang *et al*., 2019).

### Adequacy of the CMN in vitro cultivation system for Zn plant‐to‐plant transfer studies

In the present study, an *in vitro* culture system in which two *M. truncatula* plants were connected by a CMN, was used to demonstrate the plant‐to‐plant transfer of Zn. A Myc− isogenic plant was included in the Receiver‐C as control. Several fungal and plant parameters were first evaluated to ascertain the adequacy of the system. A high number of active hyphae (*c*. 276 averaged over both −Zn and +Zn treatments) crossed the partition wall separating the Donor‐C from the Receiver‐C. Mycelium developed abundantly in both compartments with hyphal length and density averaging 1878 cm and 118 cm cm^−3^, irrespective of Zn treatment. These values were almost identical to those reported by Voets and colleagues (2008) and Dupré de Boulois and colleagues (2006) in similar *in vitro* systems after respectively 10 and 12 weeks of growth. The total number of spores was much higher in the Receiver‐C as compared with the Donor‐C (8790 vs 3679 spores) under both +Zn and −Zn treatments, and these values were similar to those previously found by Voets and colleagues (2008) (6970 vs 2791 spores). These results support the fact that spore production is dependent on extraradical mycelium biomass and on the re‐allocation of resources from intraradical to extraradical hyphal mycelium. Indeed, the hyphal length and hyphal density was higher in the Receiver‐C than in the Donor‐C at both Zn treatments, although no significant differences were detected possibly due to the variability among replicates. Irrespective of Zn treatment, hyphal length per root length and hyphal length per AM fungal colonized root length were much higher in the Receiver‐C than in the Donor‐C. These differences were mainly due the differential root growth in the two compartments, according to the age of the plants and this might be the reason of the differential number of spores in the Donor‐C and Receiver‐C. AM fungal root colonization was confirmed in AM‐Donor and AM‐Receiver plants, both by the classical method of microscope counting and by the quantitative assessment using qPCR methodology. Whatever the treatment, plant root colonization of AM‐Donor and AM‐Receiver was high (in the range 45.6%–52.4%), while it was totally absent in the NM‐Control. While no difference was highlighted in percentage of colonization between AM‐Donor and AM‐Receiver under −Zn, significant difference was detected under +Zn (53% vs 46%) using microscopy. However, this was not confirmed by *Ri28S* expression that showed a high degree of correlation with the total root colonization quantified by microscopy in all plants, except for the AM‐Receivers under +Zn. It is thus probable that molecular determination is more reliable since less affected by the operator's measurement (Alkan *et al*., 2004), and consequently AM fungal colonization can be considered similar in all compartments and not affected by Zn treatment. Moreover, according to Parniske (2008), the functionality of the symbiosis was confirmed by the visualization of the arbuscules (data not shown) within the cells of the AM‐Donor and AM‐Receiver roots in both compartments.

No diffusion of medium from Donor‐C to Receiver‐C or root development in the Receiver‐C, and no microbial contamination were noticed in both compartments. Regarding plant growth parameters, Zn limitation was not detrimental to plant growth, as shown by the lack of variation in plant biomass and root length between the plants in the +Zn and –Zn treatments. There were age‐related size differences between the AM‐Donor and the AM‐Receiver, showing that the plants were growing properly, and no differences between the AM‐Receiver and NM‐Control, showing their growth balance.

### Zn is transferred from a donor plant to the root and shoot of a receiver plant linked by the CMN

Zinc transfer was demonstrated from donor to receiver plants as earlier demonstrated for ^13^C from shoot of a donor plant to roots of a receiver plant (Voets *et al*., 2008) and for radioceasium from shoot of a donor plant to shoot of a receiver plant (Gyuricza *et al*., 2010) and very recently for warning signals in healthy plants connected to plants attacked by a fungal pathogen (Alaux *et al*., 2020), extending the transport role of CMNs to semiochemicals. Although in our system indubitable exclusion of the indirect transfer of Zn could not be ascertained, the very low amount of Zn in the Donor‐C medium (about 1 μg g^−1^ d.w.; data not shown) suggests that if an indirect transfer occur, it would account for negligible quantities. In addition, the very low amount of Zn in NM‐Control was due to the Zn released by hyphal exudation and decay in the Receiver‐C, because no root colonization by the AM fungus was noticed. The Zn released in the receiver growth medium was confirmed by the analysis of the Zn concentration in the medium (about 1 μg g^−1^ d.w.; data not shown). Quantitatively, the efficiency of Zn application was 19.8%, considering the ratio between the total Zn content in AM‐Donor, AM‐Receiver and media and the amount of Zn applied to the leaves of the AM‐Donor. Thus, about 80% of the applied Zn was not absorbed by leaves and was not released into the MSR medium (see the low amount found in the media of both compartments). However, Zn adhering to the leaf surface was carefully removed before the analytical determination, according to the procedure of sampling preparation (Yilmaz *et al*., 2017). The total Zn content of the system (19.8 μg Zn plant^−1^) was partitioned as follows: 89.9% and 8.5% into the shoots and roots of the AM‐Donor, and 1.2% and 0.3% into the roots and shoots of the AM‐Receiver. Thus, considering the percentage of Zn presents in the roots of the donor plant (i.e., 8.5%) and accessible to the AM fungus, 12.2% and 3.4% was transferred to the roots and shoots of the AM‐Receiver respectively, whereas 84.4% remained in the roots of the AM‐Donor. The Zn transfer from roots to shoots in the AM‐Receiver was expected, because it was demonstrated under a same system that P, used as tracer for studying the transport from HC to RC, is transferred from AM‐colonized receiver roots to shoots in a proportion of 20.7% of the initial P supplied in the HC (Dupré de Boulois *et al*., 2006). Compared with Zn, the larger proportion of P transferred to shoots might be determined by the major role played by P for plant physiology.

Although the proportion of Zn transferred by the CMN from the AM‐Donor to the roots and shoots of the AM‐Receiver was low, the transfer ensures a Zn concentration in the receiver plant tissues above the critical threshold for maintaining cell structure and function (15 μg Zn g^−1^ dry weight, according to Broadley *et al*., 2012). Thus, in plant communities or in agricultural systems, the CMN may modulate the transfer and allocation of Zn among plants.

### Transcriptional pattern of Zn transport‐related processes in a receiver plant linked by the CMN after Zn application to a donor plant

In the AM‐Receiver an upregulation of the *MtZIP2* gene was found in roots as compared with the NM‐Control only in the +Zn treatment. Similarly, after foliar application of Zn, *MsZIP2* was significantly upregulated in roots and shoots of *Medicago sativa* (Cardini *et al*., 2021). The change of expression found in our +Zn treatment can be interpreted as an indication of Zn storage in the xylem parenchymal cells of the AM‐Receiver roots after transfer of Zn. Indeed, in *A. thaliana*, the expression of *ZIP2* was localized in the root stele, supporting the role of ZIP2 protein in metal transport into the stele that presumably promotes the accumulation of Zn in the xylem parenchyma (Milner *et al*., 2013). Moreover, our results on the upregulation of *MtZIP2* in AM‐Receiver in comparison with NM‐Control cannot be interpreted as an indirect effect of being colonized by AMF, since this gene was significantly affected by Zn and not affected by AMF or the interaction between AMF and Zn (Watts‐Williams *et al*., 2017). These results are also supported by recent evidences that *MtZIP2* is similarly highly upregulated in inoculated and not‐inoculated *M. truncatula* plants at both low and high Zn soil availabilities (Watts‐Williams *et al*., 2020). The undetectable *MtZIP2* expression in the shoots of AM‐Donor, AM‐Receiver and NM‐Control under +Zn and −Zn treatments, is supported by the pathway of this gene in *M. sativa* and *M. truncatula* after Zn application that showed a stronger expression in roots than in shoots/stems and no expression in leaves (Burleigh *et al*., 2003; Cardini *et al*., 2021).

As regard *MtZIP1*, it was only downregulated in the shoots and roots of the AM‐Donor as compared with AM‐Receiver under Zn application, suggesting the success of the Zn application and translocation within the plant. This response is consistent with the expression of *ZIP1* when plants are in Zn deficient conditions (Eidie *et al*., 1996; Ramesh *et al*., 2003; Ishimaru *et al*., 2006). Moreover, since the ZIP proteins can transport across membranes not only Zn^2+^, but also other metals, including Cd^2+^, Fe^3+^/Fe^2*+*^, Mn^2+^, Ni^2+^, Co^2+^ and Cu^2+^ (Grotz *et al*., 1998; Eckhardt *et al*., 2001; Mäser *et al*., 2001), we can hypothesize that CMN can also be involved in the transport of other metals between plants.

Another indication of Zn transfer between plants is represented by the *MtNAS1* transcriptional response in roots and shoots of the AM‐Receiver. In the roots and shoots of these plants the upregulation of *MtNAS1* as compared with the NM‐Control in the +Zn treatment can support the increased transport of Zn in the form of the ligand complex zinc‐nicotianamine (Zn‐NA). Indeed, NA is considered a fundamental chelator for Zn sequestration in vacuoles and Zn redistribution within the plant (Deinlein *et al*., 2012). Since the nicotianamine level greatly correlates with *NAS* transcripts (Talke *et al*., 2006; Haydon *et al*., 2012), the *NAS* expression found in our system can be considered a reliable indicator of NA content and Zn chelation. Moreover, under Zn foliar application, *MtNAS1* was upregulated in AM‐Donor shoots and roots. This is similar to the expression observed for *NAS1* and *NAS2* in *M. sativa* and *Triticum durum* plants after Zn foliar application (Deshpande *et al*., 2018; Cardini *et al*., 2021). This confirms the strategy of the plants to chelate Zn in the shoot tissues and then to load it into the phloem, enabling its redistribution to roots.

Here, the fact that *MtZIP14* gene was similarly expressed in both AM‐Donor and AM Receiver under +Zn and −Zn treatments cannot be explained by the results of Watts‐Williams and colleagues (2020) reporting that *MtZIP14* is a membrane protein able to facilitate Zn transport into the root cells. Indeed, given the fact that localization and function of this protein would not change at low and high Zn availabilities, we expected an opposite pattern of the *MtZIP14* gene expression in the two compartments. Although in the AM‐Receiver the behaviour of the gene supports Zn transfer from the fungus to the plant, the inconsistency observed in the AM‐Donor plants cannot be explained by the Zn uptake from the donor medium according to recorded low concentration of Zn.

The *RiZnT1*, belonging to the CDF family, follows this pattern after foliar Zn application: upregulation in the roots of the AM‐Donor and AM‐Receiver and as expected no transcript detection in the roots of the NM‐Control. The transcriptional level of *RiZnT1* in the structures of *R. irregularis* within the roots of the AM‐Receiver supports the increased concentration of Zn in roots mediated by the CMN. The model for metal delivery by AMF to the host plants supports the localization of *RiZnT1* in the vacuolar compartments of the extraradical mycelium (González‐Guerrero *et al*., 2008). However, in our study, the fact that *RiZnT1* was expressed in the roots of both AM‐Donor and Receiver under +Zn can support a role of this gene in the vacuolar Zn sequestration also in the intraradical mycelium with vacuoles acting as carrier within the AM‐Donor roots and a possible different role in the intraradical mycelium within the roots of the AM‐Receiver (Ashford, 2002; Javot *et al*., 2007; González‐Guerrero *et al*., 2008). The *RiZnT1* can play a functional role in addition to other specific transporters identified in the arbuscules (González‐Guerrero *et al*., 2008), and might have a dual function depending on Zn availability (e.g. change in localization depending on Zn availability as suggested for some yeast Zn transporters and for the ZIP family Zn transporter SIZRT2 of the ectomycorrhizal fungus *Suillus luteus*; Coninx *et al*., 2019).

Similarly, under Zn application, the *RiZRT1*, belonging to the ZIP family, was upregulated in the roots of the AM‐Donor and AM‐Receiver. Thus, in agreement with the results on the expression of *RiZnT1*, we hypothesize a dual function of the RiZRT Zn transporter. This trait would allow to support the role played by the AM fungus in the trafficking of Zn among plants, and encourage an in‐depth functional characterization of the studied *Rhizophagus* Zn transporters to confirm our hypothesis.

Finally, the fact that both AM fungal Zn genes are not expressed in the NM‐control under both −Zn and +Zn applications additionally demonstrated that the AM fungus did not colonize the symbiosis‐defective mutant line. Moreover, the different expression profile of the fungal Zn genes in −Zn Donor and Receiver plants is likely to be due to an early fungal response to Zn limiting conditions (i.e., in the Receiver plants), while this response is not detected anymore with plant ageing.

## Conclusions

The present work is the first that provides strong evidence for a direct transfer of Zn between two plants only connected by a CMN. It further demonstrates a triggering of the transcription of fungal and plant genes involved in Zn transport‐related processes. Thus, this study increases the range of functions undertaken by CMNs. The *in vitro* cultivation system allows to study plant‐to‐plant Zn transfer in a context free of any undesirable microorganisms or other unwanted factors (e.g., soil characteristics). However, further studies are needed under pot culture conditions with plants growing in separate compartments only connected by CMN to confirm the results and ascertain the potential applicability of findings in agricultural systems for improving the redistribution of Zn among plants.

## Experimental procedures

### Biological material

*Medicago truncatula* Gaertn., cv. ‘Jemalong’ (SARDI, Australia) wild type (line J5; Myc+/Nod+) was used as Zn‐donor plant (AM‐Donor, hereafter) and Zn‐receiver plant (AM‐Receiver, hereafter). Its symbiosis‐defective mutant, line TRV25 (Myc−/Nod−) (Sagan *et al*., 1995, 1998; Morandi *et al*., 2005) was used as control plant (NM‐Control, hereafter). More details about this symbiosis‐defective mutant are given in the Method S1. The AM fungus *Rhizophagus irregularis* (Błaszk., Wubet, Renker, and Buscot) C. Walker and A. Schüßler (2010) strain MUCL 41833, was provided by the Glomeromycota *in vitro* collection GINCO (http://www.mycorrhiza.be/ginco-bel) on Ri T‐DNA transformed chicory (*Cichorium intybus* L.) roots and further sub‐cultured following Cranenbrouck and colleagues (2005).

Seeds of *M. truncatula* were surface‐sterilized and placed in Petri plates (92 mm diameter, 10 seeds per plate) on the Modifed Strullu–Romand (MSR) medium (Declerck *et al*., 1998), lacking sucrose, vitamins and Zn (MSR −Zn), and solidified with 3 g L^−1^ of Gellan Gum (Alfa Aesar, Karlsruhe, Germany) (thickness of 0.5 cm). The Petri plates were incubated in the dark at 27°C.

### Experimental set‐up

The experimental system consisted in bi‐compartmented Petri plates, with a donor (Donor‐C) and receiver (Receiver‐C) compartment, each containing one autotrophic *M. truncatula* plant (J5 line) linked by a CMN and, for the Receiver‐C only, a mutant plant (TRV25 line) used as control (see Voets *et al*., 2008). In the Donor‐C, a hole was made to insert the AM‐Donor J5 plant, while in the Receiver‐C two holes were made to insert the AM‐Receiver J5 and the NM‐Control TRV25 plants (Fig. 4). The Donor‐C and the Receiver‐C were filled with 30 ml of MSR ‐Zn medium.

In each Petri plate, a 5‐day old *M. truncatula* seedling was inserted into the Donor‐C with the roots plated on the medium and shoot extending outside the plate via the hole. The roots were inoculated after 2 weeks with about 100 spores of *R. irregularis* following Cranenbrouck and colleagues (2005). Details about how the plants were inoculated and the growth conditions are given in Methods S2. Three weeks after inoculation, 10 ml of sterilized MSR −Zn medium was added in the Donor‐C. This addition was repeated every 2 weeks throughout the experiment. Roots were regularly trimmed to avoid crossing the partition wall. Eight weeks after inoculation, a profuse mycelium was established in the Donor‐C that crossed the partition wall and developed abundantly in the Receiver‐C. At that time, the AM‐Receiver and the NM‐Control plants (5‐day old seedlings) were inserted in the Receiver‐C, following the same procedure as above.

### Zinc foliar application and sampling

Zinc was applied to the leaves of the AM‐Donor plant at 0.25 g Zn L^−1^ as ZnSO_4_·7H_2_O (+Zn treatment). A water control (i.e., −Zn treatment) was also included. A total of 34 Petri plates were set up with 17 replicates per treatment (17 for +Zn and 17 for −Zn treatment). The pH of both solutions was adjusted to 6.2. A drop of Tween® 20 (Sigma‐Aldrich, Steinheim, Germany; 10 μl) was added to both solutions to increase the adhesion to the leaves. The solutions were applied to the middle laminae of all leaves of the AM‐Donor plants as 10 droplets (10‐μl each) in four applications at 24 h intervals (at 0, 24, 48 and 72 h) (total volume of 400 μl per plant), corresponding to the doses of 0.1 or 0 mg of Zn per plant.

The adequate time of Zn application/sampling was determined as described in Methods S2. The application of Zn or water solutions was done at week 16 from the plating of the AM‐Donor plants in the Donor‐C (plants were still at vegetative growth stage and any symptoms of nutrient deficiencies was detected), that corresponds to 8 weeks after having insert the AM‐Receiver plants in the Receiver‐C. Five days later, shoots and roots of plants in the DC and RC were sampled for further analysis, and the plates were used to measure the fungal mycelium parameters.

### Plant and AM fungus morphological measures

At harvest, the number of leaves, stem and roots lengths of AM‐Donor, AM‐Receiver and NM‐Control plants were assessed on five plates, randomly chosen out of the 17 plates per treatment. Root length was estimated by the gridline intersect method (Newman, 1966). Shoot dry weight was determined after oven drying at 70°C to constant weight. Root fresh weight was measured and root dry weight was determined on a subsample. An additional subsample of roots was soaked in water and checked for the presence on root surface of extraradical hyphae and spores, which were carefully plucked with forceps under a dissecting microscope and successively used for RNA extraction. The roots were cleared in 10% KOH solution at 90°C for 5 min, acidified in HCl 2% for 10 min and stained with 0.05% Trypan blue, using lactic acid instead of phenol (Phillips and Hayman, 1970), at 90°C for 5 min, and percentage of colonization assessed using the gridline intersect method (Giovannetti and Mosse, 1980). The AM fungal colonized root length was calculated multiplying the root length by the percentage of colonization. The total hyphal length was measured in both compartments (after root removal) using the *HyLength* image analysis tool (Cardini *et al*., 2020). The hyphal density in the Donor‐C and Receiver‐C was calculated as mean of measures taken from 10 randomly acquired images (20 mm^2^) in different areas per compartment. Details about images and calculations of hyphal traits are given in Methods S2. The number of active hyphae (i.e., presenting bidirectional flux of cytoplasm/protoplasm) crossing the partition wall was measured under a stereomicroscope (×40). The spore number was assessed in both compartments, following the method of Voets and colleagues (2005).

### Plant Zn analysis

The 12 remaining plates of each treatment were used for the Zn concentration analysis. To obtain enough material for Zn analysis four randomly chosen AM‐Donor, AM‐Receiver and NM‐Control plants were merged and thus three replicates were analysed for each treatment. The shoot of each plant was rinsed in a 1 mM CaCl_2_ solution to remove the Zn adhering to the surface (Yilmaz *et al*., 2017). Zinc concentration was determined by inductively coupled plasma optical emission spectroscopy (ICP‐OES) on an Optima 8000 spectrometer (Perkin Elmer, Waltham, MA, USA), following the procedure of Nölte (2003). Zinc concentration was also analysed in the MSR ‐Zn medium collected from both compartments of each treatment. However, it was not possible to analyse Zn concentration in the AM fungal mycelium of both compartments due to weight limitation.

### Selection of plant–fungal genes encoding Zn transporters and design and validation of RT‐qPCR assays

Three genes of *M. truncatula*, *MtZIP1*, *MtZIP2* and the gene encoding nicotianamine synthase, *Mt‐NAS1*, were selected from previous studies (Burleigh *et al*., 2003; López‐Millán *et al*., 2004; Curie *et al*., 2008; Clemens *et al*., 2013; Cardini *et al*., 2021) (Fig. 5). Moreover, the Zn transporter gene *MtZIP14*, localized on the PAM, was selected (Watts‐Williams *et al*., 2020). Two genes of *M. truncatula*, actin‐101 (*MtACT‐101*) and elongation factor 1‐α (*MtEF1‐α*) were selected as reference genes (Nicot *et al*., 2005). Two genes of *R. irregularis* were selected: the *RiZnT1* (formerly known as *GintZnT1* by González‐Guerrero *et al*., 2005) and the putative Zn transporter *RiZRT1* (Tamayo *et al*., 2014) (Fig. 5). The *R. irregularis* 28S ribosomal subunit (*Ri28S*) was used as reference gene (Alkan *et al*., 2004).

The designed *Medicago sativa* qPCR primers, targeting *ZIP1*, *ZIP2* and *NAS1* and the two reference genes *ACT‐101* and *EF1‐α*, were validated in *M. truncatula*, using the procedure described by Cardini and colleagues (2021). Similarly, the primers designed by Watts‐Williams and colleagues (2020) and targeting *ZIP14* were validated. Details about qPCR primers and validation are given in Tables S7 and S8, and Methods S3. BLAST of the sequences of amplicons obtained by PCR using the six sets of primers proved the specificity of the assays. Specificity was confirmed by the analysis of melting curves of each qPCR amplicon, which did not show non‐specific amplification products in dissociation (Fig. S2). Very strong precision of the qPCR assays was demonstrated by the coefficients of determination (R^2^) of the standard curves (*R* ^2^: ≥ 0.99; Table S7; Fig. S3), while the high accuracy was proven by the efficiency (E ≥ 99.3%).

Two new RT‐qPCR assays were developed for *R. irregularis*. Details about primer design and test and PCR and qPCR conditions are given in Tables S5, S7 and Methods S4. Similarly to plant primers, primer specificity was proven by BLAST and confirmed by the analysis of melting curves (Fig. S2). A very strong precision and high accuracy was found (*R* ^2^ ≥ 0.99; E ≥ 97.9%; Table S7; Fig. S3).

### RNA isolation and Real‐Time RT‐PCR

Total RNA was extracted from 50 mg of representative subsamples of shoot tissues and from 50 mg of representative subsamples of root tissues of AM‐Donor, AM‐Receiver and NM‐Control plants, using the RNeasy Mini Kit (Qiagen, Hilden, Germany). A total of 60 RNA extractions were performed, five replicates for each organ (shoot and root) of three types of plants (AM‐Donor, AM‐Receiver and NM‐Control) per treatment. For additional details see Methods S5. From each RNA extract, 1 μg of total RNA was reverse transcribed to complementary DNA (cDNA) using the iScript cDNA Synthesis Kit (Biorad, Hercules, California) and the RT‐qPCR assays (Table S7) were run in 20 μl reaction volume, as described in Methods S5. The two RT‐qPCR assays (*RiZnT1* and *RiZRT1*; Table S7) were applied only on cDNA of the root samples. Three technical replicates were run for each cDNA sample (the mean of the three technical replicates for each of the five biological replicates were handled in the statistical analyses). The qPCR conditions are given in Methods S5. The transcript levels of *MtACT‐101* and *MtEF1‐α* were determined on all cDNA samples (Nicot *et al*., 2005) and then used as normalization controls, since they did not vary following Zn application. The transcript levels of the *Ri28S* of *R. irregularis* were determined on all cDNA root samples (Alkan *et al*., 2004) and then used for normalization. The relative expression analysis of all genes was done by the double standardization method (ΔΔCq) that requires the reference genes and the control treatment (Livak and Schmittgen, 2001; Vandesompele *et al*., 2002). The transcript level of the plant genes in the NM‐Controls (mean of the NM‐Controls in +Zn and −Zn treatment) was used as control, whereas the transcript level of the fungal genes in the AM‐Receiver roots in the −Zn treatment was used as control.

### Statistical analyses

To verify the transfer of Zn between Zn treated AM‐Donor plants and AM‐Receiver plants (Hypotheses 1 and 2) we set up a single factor design with plant type (AM‐Donor, AM‐Receiver and NM‐Control) as independent variable and Zn concentration and plant gene expression as dependent variables. Data were analysed by one‐way ANOVA and orthogonal contrasts, such as AM‐Receiver versus NM‐Control (Hypothesis 1) and AM‐Donor versus AM‐Receiver (Hypothesis 2) plants. Additionally, to verify that the transfer mediated by CMN does not occur in absence of Zn application (Hypothesis 3), the same experimental design and statistical approach on Zn concentration and gene expression was applied under no‐Zn application (one‐way ANOVA and orthogonal contrasts). To further support the evidence of Zn transfer from AM‐Donor to AM‐Receiver plants, pairwise comparisons of Zn‐fertilized and unfertilised plants were performed on Zn concentration by *t*‐test for AM‐Donor, AM‐Receiver and NM‐Control plants, and on fungal gene expression for AM‐Donor and AM‐Receiver.

To verify the adequacy of the system under Zn‐ and no‐Zn application we applied a single factor design with plant type (AM‐Donor, AM‐Receiver and NM‐Control) as independent variable and plant growth traits as dependent variables. Data were analysed by one‐way ANOVA and orthogonal contrasts, such as AM‐Receiver versus NM‐Control and AM‐Donor versus AM‐Receiver plants. Moreover, fungal traits in Donor‐C and Receiver‐C were analysed by *t*‐tests, under both Zn‐ and no‐Zn application. To verify the adequacy of the system excluding any toxic effect on plants, we analysed the effect of Zn on plant growth traits by *t*‐test, separately for AM‐Donor, AM‐Receiver and NM‐Control plants. Finally, to verify the adequacy of the system excluding any toxic effect on fungus, we analysed the effect of Zn on fungal traits by *t*‐test, separately for Donor‐C and Receiver‐C.

Data were transformed when needed to fulfil the assumptions of the ANOVA. All the analyses were performed using the SPSS software package version 21.0 (SPSS, Chicago, IL, USA).

## Author contributions

Elisa Pellegrino and Laura Ercoli conceived the ideas and designed the methodology; Stéphane Declerck designed the *in vitro* system; Alessio Cardini and Maryline Calonne‐Salmon performed *in vitro* culturing, microscopy and staining analysis; Alessio Cardini and Barbara Mazzolai designed and performed real‐time PCR; Alessio Cardini and Elisa Pellegrino performed chemical analyses; Alessio Cardini and Elisa Pellegrino performed data analysis; Alessio Cardini, Elisa Pellegrino and Laura Ercoli led the writing of the article; all authors contributed critically to the drafts and gave final approval for publication.

## Supporting information


**Fig. S1.** Transcript levels of the *Ri28S* in the roots of the AM‐Donors and Receivers *Medicago truncatula* plants colonized by *Rhizophagus irregularis* under +Zn and −Zn conditions.
**Fig. S2.** Melting peaks of qPCR products obtained with primers targeting *Medicago truncatula* genes (*MtZIP1*, *MtZIP2*, *MtZIP14*, *MtNAS1*, *MtACT‐101* and *MtEF1‐α*) and *Rhizophagus irregularis* genes (*RiZnT1*, *RiZRT1*, *Ri28S*).
**Fig. S3.** Standard curves of qPCR products obtained with primers targeting *Medicago truncatula* genes (*MtZIP1*, *MtZIP2*, *MtZIP14*, *MtNAS1*, *MtACT‐101* and *MtEF1‐α*) and *Rhizophagus irregularis* genes (*RiZnT1*, *RiZRT1*, *Ri28S*).
**Table S1.** Growth parameters of the *Medicago truncatula* AM‐Donor, AM‐Receiver and NM‐Controls under +Zn and –Zn conditions.
**Table S2.** *P* values of the *t*‐tests on the effect of Zn foliar application on plant growth parameters, AMF root colonization and shoot and root Zn concentrations of AM‐Donor, AM‐Receiver and NM‐Control *Medicago truncatula* plants.
**Table S3.** *P* values of the *t*‐tests on the effect of Zn foliar application on AMF extraradical mycelium growth parameters and spore number in donor and receiver compartments.
**Table S4.** Zinc concentrations in shoots and roots of AM‐Donor, AM‐Receiver and NM‐Control *Medicago truncatula* plants under +Zn and –Zn conditions.
**Table S5.** *P* values of the linear orthogonal contrasts on the relative gene expressions of *MtZIP1*, *MtZIP2*, *MtZIP14*, *MtNAS1* in shoots and roots, and on the fungal relative gene expressions of *RiZnT1* and *RiZRT1* in AMF colonized roots under +Zn and –Zn conditions.
**Table S6.** *P* values of *t*‐test on the relative gene expressions of *MtZIP1*, *MtZIP2*, and *MtNAS1* in shoots and roots, and of *MtZIP14*, *RiZnT1* and *RiZRT1* in AM fungal colonized roots under +Zn and –Zn conditions.
**Table S7.** Sequences of the qPCR primer pairs used in the study and parameters of the validation.
**Table S8.** The *Medicago truncatula* and *Rhizophagus irregularis* sequences used to design the qPCR primers by the Primer‐Blast online tool in NCBI.
**Methods S1.** Description of the mycorrhizal defective mutant isogenic line TRV25.
**Methods S2.** Set‐up, such as plant growth conditions and time of Zn application/sampling and details on fungal measures.
**Methods S3.** Details on RT‐qPCR validation of the *MtZIP1*, *MtZIP2* and *MtNAS1* primers.
**Methods S4.** Details on RT‐qPCR primer design for the *Rhizophagus irregularis* genes *RiZnT1* and *RiZRT1* and validation of the new RT‐qPCR assays.
**Methods S5.** Details on RNA isolation and real‐time RT‐qPCRs.
