# Supplementary material for: Direct transfer of zinc between plants is channelled by common mycorrhizal network of arbuscular mycorrhizal fungi and evidenced by changes in expression of zinc transporter genes in fungus and plant
Source: Environ Microbiol. 2021 May 10;23(10):5883–900. doi: 10.1111/1462-2920.15542 (PMC8597171; doi:10.1111/1462-2920.15542)
Supplement: Supplementary file 1 — Fig. S1. Transcript levels of the Ri28S in the roots of the AM‐Donors and Receivers Medicago truncatula plants colonized by Rhizophagus irregularis under +Zn and −Zn conditions. Fig. S2. Melting peaks of qPCR products obtained with primers targeting Medicago truncatula genes (MtZIP1, MtZIP2, MtZIP14, MtNAS1, MtACT‐101 and MtEF1‐α) and Rhizophagus irregularis genes (RiZnT1, RiZRT1, Ri28S). Fig. S3. Standard curves of qPCR products obtained with primers targeting Medicago truncatula genes (MtZIP1, MtZIP2, MtZIP14, MtNAS1, MtACT‐101 and MtEF1‐α) and Rhizophagus irregularis genes (RiZnT1, RiZRT1, Ri28S). Table S1. Growth parameters of the Medicago truncatula AM‐Donor, AM‐Receiver and NM‐Controls under +Zn and –Zn conditions. Table S2. P values of the t‐tests on the effect of Zn foliar application on plant growth parameters, AMF root colonization and shoot and root Zn concentrations of AM‐Donor, AM‐Receiver and NM‐Control Medicago truncatula plants. Table S3. P values of the t‐tests on the effect of Zn foliar application on AMF extraradical mycelium growth parameters and spore number in donor and receiver compartments. Table S4. Zinc concentrations in shoots and roots of AM‐Donor, AM‐Receiver and NM‐Control Medicago truncatula plants under +Zn and –Zn conditions. Table S5. P values of the linear orthogonal contrasts on the relative gene expressions of MtZIP1, MtZIP2, MtZIP14, MtNAS1 in shoots and roots, and on the fungal relative gene expressions of RiZnT1 and RiZRT1 in AMF colonized roots under +Zn and –Zn conditions. Table S6. P values of t‐test on the relative gene expressions of MtZIP1, MtZIP2, and MtNAS1 in shoots and roots, and of MtZIP14, RiZnT1 and RiZRT1 in AM fungal colonized roots under +Zn and –Zn conditions. Table S7. Sequences of the qPCR primer pairs used in the study and parameters of the validation. Table S8. The Medicago truncatula and Rhizophagus irregularis sequences used to design the qPCR primers by the Primer‐Blast online tool in NCBI. Methods S1. [file EMI-23-5883-s001.docx]

**Direct zinc transfer between plants is channelled by common mycorrhizal network of arbuscular mycorrhizal fungi and evidenced by changes in expression of zinc transporter genes in fungus and plant**

Alessio Cardini^1†^, Elisa Pellegrino^1†*^, Stéphane Declerck^2^, Maryline Calonne-Salmon^2^, Barbara Mazzolai^3^, Laura Ercoli

^1^ Institute of Life Sciences, Sant’Anna School of Advanced Studies, P.za Martiri della Libertà 33, 56127 Pisa, Italy.

^2^ Université catholique de Louvain, Earth and Life Institute, Applied Microbiology, Mycology, Croix du Sud 2, box L7.05.06, 1348, Louvain-la-Neuve, Belgium.

^3^ Center for Micro-BioRobotics, Istituto Italiano di Tecnologia, Viale Rinaldo Piaggio 34, 56025 Pontedera, Pisa, Italy.

*For correspondence. Elisa Pellegrino, E-mail [elisa.pellegrino@santannapisa.it](mailto:elisa.pellegrino@santannapisa.it); Tel (+39) 050 883181.

^†^These authors contributed equally to this work.

**
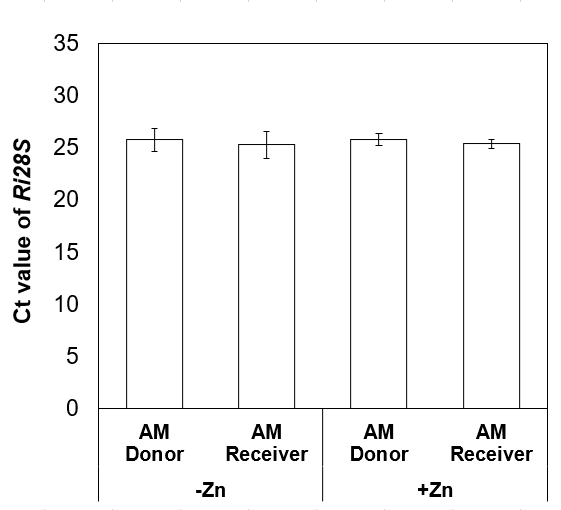
**

**Fig. S1.** Transcript levels of the *Ri28S* in the roots of the AM-Donors and Receiver plants of *Medicago truncatula* Gaertn., cv. ‘Jemalong’ wildtype (line J5; Myc+/Nod+), colonized by *Rhizophagus irregularis* isolate MUCL 41833 under +Zn and -Zn conditions. The Zn solutions were applied to the leaves of the AM-Donor plants at the doses of 0 and 0.1 mg of Zn plant^-1^ (+Zn and –Zn, respectively). The plants were grown in the autotrophic *in vitro* setup described by Voets *et al*. (2008).

**Fig. S2.** Melting peaks of the qPCR products obtained with the primers targeting the *Medicago truncatula* genes, *MtZIP1*, *MtZIP2*, *MtZIP14* and *MtNAS1*, and the two reference genes *MtACT-101* and *MtEF1-α*. Melting peaks of the qPCR products obtained by the newly designed primers targeting *Rhizophagus irregularis* genes, *RiZnT1* and *RiZRT1*, and by the primers developed by Alkan et al. (2004), targeting the fungal reference gene *Ri28S*.

**
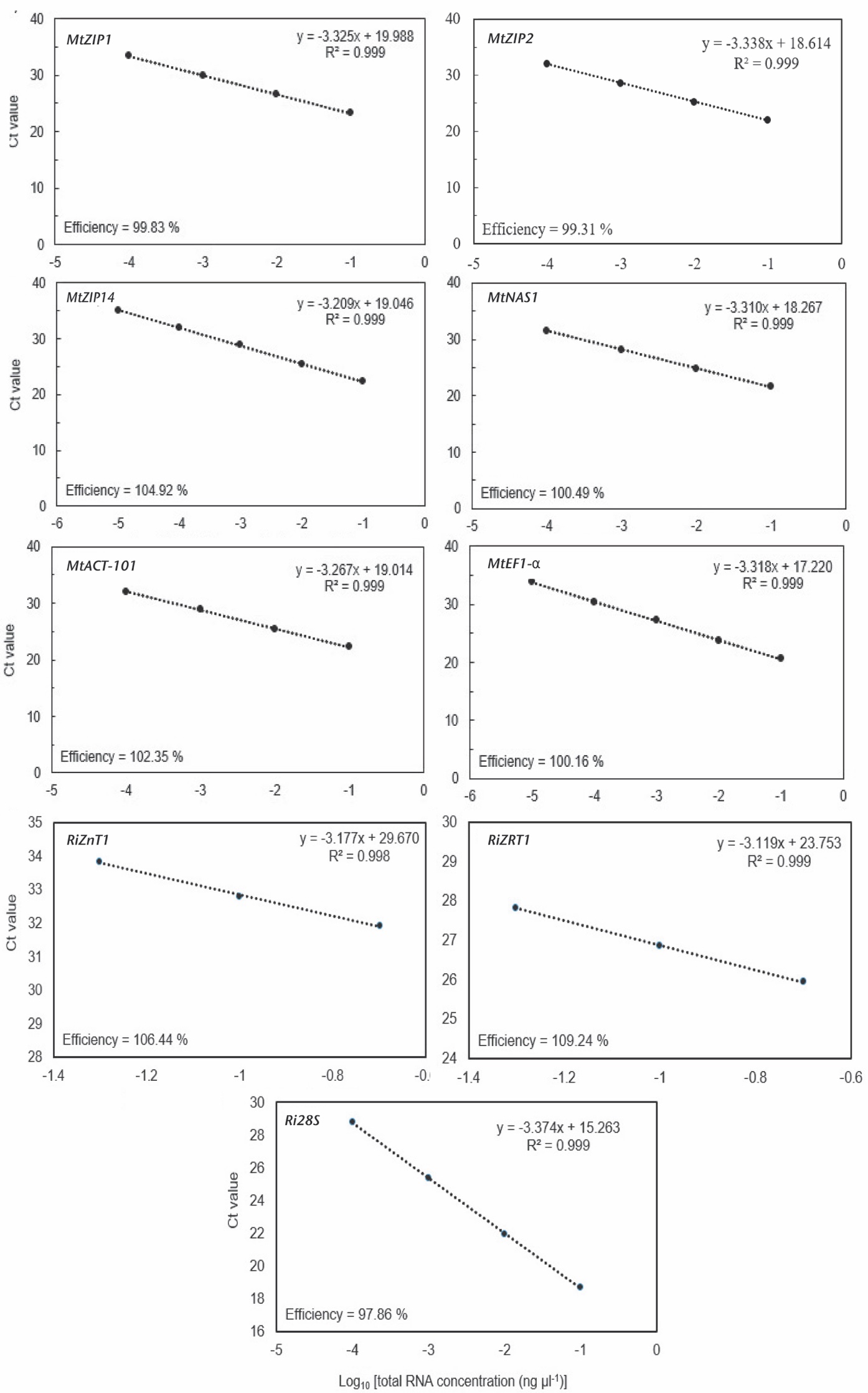
**

**Fig. S3**. Standard curves of qPCR using five primer pairs targeting the *Medicago truncatula* genes, *MtZIP1*, *MtZIP2*, *MtZIP14*, *MtNAS1*, and the two reference genes *MtACT-101* and *MtEF1-α*. Standard curves of the three primer pairs targeting the *Rhizophagus irregularis* genes, *RiZnT1* and *RiZRT1*, and the fungal reference gene *Ri28S*. The curves are based on three replicates of 10-fold serial dilutions of complementary DNA (cDNA) of *M. truncatula* and of 5-fold serial dilutions of cDNA of *R. irregularis*. The cycle threshold values (Ct) are plotted against the decimal logarithms of the concentrations of the reverse transcribed total RNA. Linear regressions are shown in each subfigure in order to indicate the linear range of quantification and the precision (R^2^). The amplification efficiency (Efficiency), as inferred from the slope of the lines (S: angular coefficient), is indicated at the bottom left of each subfigure (%).

**References**

Alkan, N., Gadkar, V., Coburn, J., Yarden, O., and Kapulnik, Y. (2004) Quantification of the arbuscular mycorrhizal fungus *Glomus intraradices* in host tissue using real‐time polymerase chain reaction. New Phytol 161: 877-885.

Voets, L., Goubau, I., Olsson, P.A., Merckx, R., and Declerck, S. (2008) Absence of carbon transfer between *Medicago truncatula* plants linked by a mycorrhizal network, demonstrated in an experimental microcosm. FEMS Microbiol Ecol 65: 350-360.

| **Table S1.** Stem length (cm), number of leaves (N° leaves) plant^-1^, shoot dry weight (SDW – mg plant^-1^) and root dry weight (RDW – mg plant^-1^) of of 16-weeks-old wild type *Medicago truncatula* donor plants (AM-Donor; Myc+/Nod+), six-weeks-old wild type receiver *M. truncatula* plants (AM-Receiver; Myc+/Nod+) and its isogenic mycorrhiza defective mutant control plants (NM-Control; Myc-/Nod-), five days after foliar zinc (Zn) application of 0 and 0.1 mg plant^-1^ (-Zn and +Zn, respectively) on the leaves of the AM-Donor plants. The AM-Donor and AM-Receiver plants were linked by the extraradical mycelium of the AM fungus *Rhizophagus irregularis* (MUCL 41833)*.* | | | | |
| --- | --- | --- | --- | --- |
| Plant^*^ | Stem length | N° leaves | SDW | RDW |
|  | cm | N° plant^-1^ | mg plant^-1^ | mg plant^-1^ |
| AM-Donor +Zn | 9.82 ± 0.62 | 21.80 ± 0.97 | 42 ± 4 | 71 ± 8 |
| AM-Receiver +Zn | 5.04 ± 0.29 | 7.60 ± 0.40 | 14 ± 1 | 12 ± 1 |
| NM-Control +Zn | 4.46 ± 0.32 | 6.60 ± 0.51 | 12 ± 1 | 9 ± 2 |
| AM-Donor -Zn | 10.66 ± 0.61 | 19.60 ± 1.29 | 49 ± 2 | 54 ± 2 |
| AM-Receiver -Zn | 5.10 ± 0.33 | 8.60 ± 0.60 | 14 ± 1 | 15 ± 1 |
| NM-Control -Zn | 4.86 ± 1.00 | 7.40 ± 0.81 | 14 ± 1 | 15 ± 2 |
| *Treatments compared (*P*-values of linear orthogonal contrasts)* | | | | |
| AM-Donor *vs* AM-Receiver +Zn | **<0.001** | **<0.001** | **<0.001** | **<0.001** |
| AM-Receiver *vs* NM-Control +Zn | :0.368 | .0.314 | .0.625 | .0.601 |
| AM-Donor *vs* AM-Receiver -Zn | **<0.001** | **<0.001** | **<0.001** | **<0.001** |
| AM-Receiver *vs* NM-Control -Zn | :0.768 | .0.387 | .0.876 | .0.850 |
| *AM-Donor: *Medicago truncatula* Gaertn., cv. ‘Jemalong’ wildtype (line J5; Myc+/Nod+); AM-Receiver: *M. truncatula* Gaertn., cv. ‘Jemalong’ wildtype (line J5; Myc+/Nod+); NM-Control: *M. truncatula* Gaertn., cv. ‘Jemalong’ mutant line TRV 25 (Myc-/Nod-). One-way analysis of variance (ANOVA) was performed to test the effect of plant type and orthogonal contrasts were used to discriminate the differences between AM-Donor and AM-Receiver plants and between AM-Receiver and NM-Control plants. Values are means ± standard error of five replicates. | | | | |

| **Table S2.** *P* values of t-tests on the effect of zinc (Zn) foliar application of 0 and 0.1 mg plant^-1^ on plant traits, arbuscular mycorrhizal (AM) fungal colonization and colonized root length and Zn concentrations in shoot and root of 16-weeks-old wild type *Medicago truncatula* donor plants (AM-Donor; Myc+/Nod+), six-weeks-old wild type receiver *M. truncatula* plants (AM-Receiver; Myc+/Nod+) and its isogenic mycorrhiza defective mutant control plants (NM-Control; Myc-/Nod-), five days after the Zn application on the leaves of the AM-Donor plants. The AM-Donor and AM-Receiver plants were linked by the extraradical mycelium of the AM fungus *Rhizophagus irregularis* (MUCL 41833)*.* | | | | | | | | | |
| --- | --- | --- | --- | --- | --- | --- | --- | --- | --- |
| Plant type | Stem length | N° leaves | Shoot dry weight | Root dry weight | Root length | AM fungal root colonization | AM fungal colonized root length | Shoot Zn concentration | Root Zn concentration |
| AM-Donor^*^ | 0.363 | 0.210 | 0.152 | 0.072 | 0.984 | 0.098 | 0.090 | **0.011** | **0.048** |
| AM-Receiver | 0.895 | 0.203 | 0.715 | 0.111 | 0.527 | 0.615 | 0.734 | **0.033** | **0.050** |
| NM-Control | 0.490 | 0.428 | 0.212 | 0.211 | 0.943 | - | - | 0.733 | 0.344 |
| *AM-Donor: *Medicago truncatula* Gaertn., cv. ‘Jemalong’ wildtype (line J5; Myc+/Nod+); AM-Receiver: *M. truncatula* Gaertn., cv. ‘Jemalong’ wildtype (line J5; Myc+/Nod+); NM-Control: *M. truncatula* Gaertn., cv. ‘Jemalong’ mutant line TRV 25 (Myc-/Nod-). | | | | | | | | | |

| **Table S3.** *P* values of t-tests on the effect of zinc (Zn) foliar application of 0 and 0.1 mg plant^-1^ on the arbuscular mycorrhizal fungal traits in each compartment (Donor-C and Receiver-C, respectively), five days after the Zn application on the leaves of the AM-Donor plants. The AM-Donor and AM-Receiver plants were linked by the extraradical mycelium of the AM fungus *Rhizophagus irregularis* (MUCL 41833)*.* | | | | | |
| --- | --- | --- | --- | --- | --- |
| Compartment^*^ | Hyphal length | N° spores | Hyphal length per root length | Hyphal length per AMF root length | Hyphal density |
| Donor-C | 0.850 | 0.544 | 0.860 | 0.412 | 0.850 |
| Receiver-C | 0.615 | 0.317 | 0.427 | 0.402 | 0.615 |
| *In the Donor-C the *Medicago truncatula* Gaertn., cv. ‘Jemalong’ wildtype (line J5; Myc+/Nod+) was grown; in the Receiver-C: the *M. truncatula* Gaertn., cv. ‘Jemalong’ wildtype (line J5; Myc+/Nod+) and the NM-Control *M. truncatula* Gaertn., cv. ‘Jemalong’ mutant line TRV 25 (Myc-/Nod-) were grown. | | | | | |

|  |  |  |
| --- | --- | --- |
| **Table S4.** Shoot and root Zn concentration (µg g^-1^) of 16-weeks-old wild type *Medicago truncatula* donor plants (AM-Donor; Myc+/Nod+), six-weeks-old wild type receiver *M. truncatula* plants (AM-Receiver; Myc+/Nod+) and its isogenic mycorrhiza defective mutant control plants (NM-Control; Myc-/Nod-), five days after foliar zinc (Zn) application of 0 and 0.1 mg plant^-1^ (-Zn and +Zn, respectively) on the leaves of AM-Donor plants. The AM-Donor and AM-Receiver plants were linked by the extraradical mycelium of the arbuscular mycorrhizal (AM) fungus *Rhizophagus irregularis* (MUCL 41833)*.* | | |
| Plant^*^ | Shoot Zn concentration | Root Zn concentration |
|  | μg g^-1^ | μg g^-1^ |
| AM-Donor +Zn | 423.3 ± 90.3 | 23.8 ± 2.3 |
| AM-Receiver +Zn | 16.8 ± 0.6 | 29.7 ± 3.2 |
| NM-Control +Zn | 13.9 ± 0.8 | 12.4 ± 3.6 |
| AM-Donor -Zn | 14.9 ± 1.0 | 16.2 ± 1.4 |
| AM-Receiver -Zn | 12.4 ± 1.2 | 20.7 ± 0.1 |
| NM-Control -Zn | 15.2 ± 3.3 | 16.6 ± 1.6 |
| *Treatments compared (*P*-values of linear orthogonal contrasts)* | | |
| AM-Donor *vs* AM-Receiver +Zn | **0.046** | 0.225 |
| AM-Receiver *vs* NM-Control + Zn | **0.050** | **0.008** |
| AM-Donor *vs* AM-Receiver -Zn | 0.443 | 0.082 |
| AM-Receiver *vs* NM-Control - Zn | 0.401 | 1.123 |
| *AM-Donor: *Medicago truncatula* Gaertn., cv. ‘Jemalong’ wildtype (line J5; Myc+/Nod+); AM-Receiver: *M. truncatula* Gaertn., cv. ‘Jemalong’ wildtype (line J5; Myc+/Nod+); NM-Control: *M. truncatula* Gaertn., cv. ‘Jemalong’ mutant line TRV 25 (Myc-/Nod-). One-way analysis of variance (ANOVA) was performed to test the effect of plant type and orthogonal contrasts were used to discriminate the differences between AM-Donor and AM-Receiver plants and between AM-Receiver and NM-Control plants. Values are means ± standard error of three replicates. | | |

| **Table S5.** *P* values of linear orthogonal contrasts on shoot and root relative gene expression of *MtZIP1, MtZIP2* and *MtNAS1* and on fungal relative gene expression of *RiZnT1* and *RiZRT1*) of 16-weeks-old wild type *Medicago truncatula* donor plants (AM-Donor; Myc+/Nod+), six-weeks-old wild type receiver *M. truncatula* plants (AM-Receiver; Myc+/Nod+) and its isogenic mycorrhiza defective mutant control plants (NM-Control; Myc-/Nod-)*.* Relative gene expression was assessed five days after foliar zinc (Zn) application of 0 and 0.1 mg plant^-1^ (-Zn and +Zn, respectively) on the leaves of the AM-Donor plants. The AM-Donor and AM-Receiver plants were linked by the extraradical mycelium of the AM fungus *Rhizophagus irregularis* (MUCL 41833)*.* | | | | | | | | |
| --- | --- | --- | --- | --- | --- | --- | --- | --- |
| Plant^*^ | Relative expression *MtZIP1* | | | Relative expression *MtZIP2* | | Relative expression | Relative expression | |
|  |  |  |  |  |  | *MtZIP14* | *MtNAS1* | |
|  | Shoot | | Root | Shoot | Root | Root | Shoot | Root |
| AM-Donor *vs* AM-Receiver +Zn | | **0.046** | **0.015** | - | 0.742 | 0.731 | **0.020** | 0.699 |
| AM-Receiver *vs* NM-Control + Zn | | 0.445 | 0.752 | - | **0.049** | **0.002** | **0.049** | **0.047** |
| AM-Donor *vs* AM-Receiver -Zn | | 0.866 | 0.608 | - | 0.732 | 0.654 | 0.344 | 0.651 |
| AM-Receiver *vs* NM-Control - Zn | | 0.386 | 0.479 | - | 0.758 | **<0.001** | 0.85 | 0.613 |
| ^*^AM-Donor: *Medicago truncatula* Gaertn., cv. ‘Jemalong’ wildtype (line J5; Myc+/Nod+); AM-Receiver: *M. truncatula* Gaertn., cv. ‘Jemalong’ wildtype (line J5; Myc+/Nod+) ; NM-Control: *M. truncatula* Gaertn., cv. ‘Jemalong’ mutant line TRV 25 (Myc-/Nod-). | | | | | | | | |

| **Table S6** |  |  |  |  |  |  |  |  |  |
| --- | --- | --- | --- | --- | --- | --- | --- | --- | --- |
| *P* values of *t*-tests on the effect of zinc (Zn) foliar application of 0 and 0.1 mg plant^-1^ (-Zn and +Zn, respectively) on the relative expression of plant and fungal genes in 16-weeks-old wild type *Medicago truncatula* donor plants (AM-Donor; Myc+/Nod+), six-weeks-old wild type receiver *M. truncatula* plants (AM-Receiver; Myc+/Nod+) and its isogenic mycorrhiza defective mutant control plants (NM-Control; Myc-/Nod-), five days after the Zn application on the leaves of the AM-Donor plants. The AM-Donor and AM-Receiver plants were linked by the extraradical mycelium of the AM fungus *Rhizophagus irregularis* (MUCL 41833)*.* | | | | | | | | | |
| Plant type | Relative expression *MtZIP1* | | Relative expression *MtZIP2* | | Relative expression *MtNAS1* | | Relative expression *the* | Relative expression *RiZnT1* | Relative expression *RiZRT1* |
|  | Shoot | Root | Shoot | Root | Shoot | Root | Root | Root | Root |
| AM-Donor | **0.047** | **0.015** | - | 0.185 | **0.007** | **0.046** | **0.040** | **0.044** | **0.029** |
| AM-Receiver | 0.843 | 0.314 | - | **0.042** | **0.044** | **0.044** | 0.301 | **0.006** | **0.050** |
| NM-Control | 1 | 0.996 | - | 0.798 | 1 | 0.993 | - | - | - |
| ^*^AM-Donor: *Medicago truncatula* Gaertn., cv. ‘Jemalong’ wildtype (line J5; Myc+/Nod+); AM-Receiver: *M. truncatula* Gaertn., cv. ‘Jemalong’ wildtype (line J5; Myc+/Nod+) ; NM-Control: *M. truncatula* Gaertn., cv. ‘Jemalong’ mutant line TRV 25 (Myc-/Nod-). | | | | | | | | | |

| **Table S7.** qPCR primer pairs targeting four genes involved in Zn transport and two constitutively expressed reference genes (*MtACT-101* and *MtEF1-α*) of *Medicago truncatula*. All primers, except for *MtZIP14* designed by Watts-Williams et al. (2020), were originally designed and successfully validated in *Medicago sativa* by Cardini et al. (2020)*.* Newly designed qPCR primer pairs targeting two Zn transporter genes of *Rhizophagus irregularis* and one constitutively expressed reference gene of *R. irregularis* (28S ribosomal subunit; *Ri28S*) gene (Alkan et al., 2004). Target organisms and genes, primer pair sequences (forward and reverse), melting temperature (°C), product size (bp), efficiency (%) and coefficient of determination of the standard curve (R^2^) of the primers are reported. The plant primer pairs were validated on a mixed sample of shoot and root cDNA of *M. truncatula* using three replicates, while the fungal primer pairs on cDNA samples of roots of *M. truncatula* colonized by *R. irregularis* using three replicates. The fungal primers were designed with Primer-Blast, NCBI. Melting temperature (°C), product size (bp), efficiency (%) and coefficient of correlation of the standard curve (R^2^) of the primers are reported. | | | | | | | | | |
| --- | --- | --- | --- | --- | --- | --- | --- | --- | --- |
| Organisms | Target gene | Forward (5'-3') | Forward Tm (°C) | Reverse (5'-3') | Reverse Tm (°C) | Product size (bp) | Efficiency (%) | | R^2^ |
| *M. truncatula* | *MtZIP1* | *ATGATTAAAGCCTTCGCGGC* | 57.3 | *TCTGCTGGAACTTGTTTAGAAGG* | 58.9 | 233 | 99.8 | 0.999 | |
|  | *MtZIP2* | *AGCCCAATTGGCGTAGGAAT* | 57.3 | *ACAGCAACACCAAAAAGCACA* | 55.9 | 215 | 99.3 | 0.999 | |
|  | *MtZIP14* | *GCATCTGCAGGGGTTCTCAT* | 66.0 | *AAGTGCTAAACTTGCCCCGA* | 66.0 | 114 | 104.9 | 0.999 | |
|  | *MtNAS1* | *GCTAGCTTGGCTGAAGATTGG* | 59.8 | *AGATACAAAGCACTCGGAGACA* | 58.4 | 87 | 100.5 | 0.999 | |
|  | *MtACT-101* | *TAACACTTCCCACTCCTCGC* | 59.4 | *CCTTCTGACCCATCCCAACC* | 57.9 | 242 | 102.4 | 0.999 | |
|  | *MtEF1-α* | *AGGAAGCTGCTGAGATGAACA* | 61.0 | *AGGATGAAACTTCCTTCACGAT* | 59.4 | 399 | 100.2 | 0.999 | |
| *R. irregularis* | *RiZnT1* | *TCACGAAACTGCTTGCTTGC* | 57.3 | *TTTGGTGGTTGTGATACCGGA* | 57.9 | 233 | 106.4 | 0.998 | |
|  | *RiZRT1* | *TGGTACCGGGGTTATTGTGG* | 59.4 | *ATGTTGCTGCCATCATTGCG* | 57.3 | 87 | 109.2 | 0.999 | |
|  | *Ri28S* | *GCTCTGGTGCCGAAAGCTT* | 58.8 | *TAACCCGTTCTAACCTATTGACCAT* | 59.7 | 64 | 97.9 | 0.999 | |

| **Table S8**. NCBI accession numbers of the sequences of three genes involved in Zn transport and two constitutively expressed reference genes (*MtACT-101* and *MtEF1-α*) of *Medicago truncatula* and NCBI accession numbers of the two Zn transporter genes of *Rhizophagus irregularis* and one constitutively expressed reference genes of *R. irregularis* (28S ribosomal subunit; *Ri28S*). These sequences were used to design the qPCR primers by Primer-Blast online tool in NCBI. | | |
| --- | --- | --- |
| Organism | Gene | Accession number |
| *M. truncatula* | *MtZIP1* | AY339054 |
|  | *MtZIP2* | AY007281 |
|  | *MtNAS* | XM_013595007.2 |
|  | *MtACT1* | XM_003593074 |
|  | *MtEF1-α* | XM_013595882.2 |
| *R. irregularis* | *RiZnT1* | XM_025331607.1 |
|  | *RiZRT1* | XM_025323242 |
|  | *Ri28S* | AJ574787.1 |

**References**

Cardini, A., Pellegrino, E., White, P., Mazzolai, B., Mascherpa, M.C., and Ercoli, L. (2021). Transcriptional regulation of genes involved in zinc uptake, sequestration and redistribution following foliar zinc application to *Medicago sativa*. Plants 10: 476. https://doi.org/10.1101/2020.05.11.088617.

Watts-Williams, S.J., Wege, S., Ramesh, S.A., Berkowitz, O., Gilliham, M., Whelan, J., and Tyerman, S.D. (2020) Identification of a unique ZIP transporter involved in zinc uptake via the arbuscular mycorrhizal fungal pathway. BioRxiv. <https://doi.org/10.1101/2020.09.28.317669>.

**Methods S1**

*Description of the mycorrhizal defective mutant isogenic line TRV25*

Sagan et al. in 1995 obtained 18 mutants after gamma-ray mutagenesis of the *Medicago truncatula* model plant. The mutant TR25 (not to be confused with the TRV25 used in this paper) showed no nodules per plant at two N doses, no acetylene reduction activity and the infection of *Glomus mosseae* was shown to be blocked by observation under microscopy. Thus, they demonstrated that the TR25 mutant is affected in its ability to develop symbiotic associations. Later, in 1998, Sagan et al. published the results on additional *M. truncatula* mutants including TRV25. This mutant was genetically analyzed and firstly classify as Nod-. However, later, Morandi *et al.* (2005) screened many mutants including TRV25 that was shown to be not colonized by several arbuscular mycorrhiza fungi (AMF) (i.e., *Glomus mosseae* and *Glomus intraradices*) also at different time after inoculation. This mutant expresses complete absence of visual nodules on the roots after inoculation with *Synorhizobium meliloti* strain 2011, when observed at 5 and 10 weeks after sowing. Moreover, Morandi *et al.* (2005) stated that “With its solid [Nod- Myc-] phenotype, TRV25 is presently a unique mutant at a locus we call Mtsym13 [previously named B (Sagan *et al.*, 1998) or Dmi3 (Catoira *et al*., 2000)]”.

**Methods S2**

*Inoculation description, growth conditions and time of Zn application/sampling*

From sub-cultures of *Rhizophagus irregularis* (Błaszk., Wubet, Renker, and Buscot) C. Walker and A. Schüßler (2010) strain MUCL 41833, we extracted a piece of gel (Gellan Gum, Alfa Aesar, Karlsruhe, Germany) containing approximately 200 spores and transferred it to an empty sterile Petri plate (diameter 9 cm) where we added 10x the volume of citrate buffer filtered through a 0.2-μm acrodisc and then sealed the plates. We agitated the Petri plate slowly on a rotating agitator (50 rotation/min) at 25–27°C for 30 min to 1h, transferred the spores attached to the extraradical mycelium to a new Petri plate containing sterile water and using a binocular microscope (under laminar flow), we separated the spores into individuals or little clusters of spores (containing 10 spores). This step was repeated 18 times in order to have the total amount of spores needed for the inoculation of the 34 Petri Plates that was 3400 (100 per plate). Thus 10 clusters of spores were placed near the growing apex of the roots of *Medicago truncatula* donor plants (in the donor compartments) where the cellular wall is thinner and easier to penetrate. After this, the Petri plates were sealed using Parafilm and sterilized silicon grease was used to plaster the holes. The Petri plates were then covered with aluminium foil, allowing the roots and AMF to grow in the dark, while the plant shoot developed in the light. The Petri plates were placed in a growth chamber (22/18°C day/night; 80% relative humidity; 16-h photoperiod; 120 µmol m-2 s-1 photosynthetic photon flux).

To determine the adequate time of Zn application/sampling in our *in vitro* system, a preliminary experiment was conducted with the set-up of following Cranenbrouck et al. (2005). Ten weeks appeared adequate to have highly colonized (>50%) donor plants together with a dense mycelium connecting the receiver plants in the receiver compartment. Six additional weeks was adequate for obtaining receiver plants with AM fungal root colonization reaching 50%. Moreover, to define the adequate time of sampling we set up a a preliminary test to evaluate the time trend of Zn transfer. In this test, Zn transfer progressively increased up to a maximum, after which did not further increase. Thus, we sampled at the time the maximum was attained (five days after Zn application to the donor plants).

*Measures*

The images for the measure of the length of the extraradical mycelium (total hyphal length) were taken at x25 magnification by a Leica DFC295 camera, mounted on a stereomicroscope (Leica M205 A, Leica microsystems, Switzerland). The Z-stack methodology in the Leica Application Suite v.3.6.0 (Leica microsystems, Switzerland) was applied for the measurements.

The total hyphal length per compartment was calculated by multiplying the hyphal density by the volume of the respective compartment. The hyphal length per root length was calculated by dividing the total hyphal length for each compartment by the corresponding root length. The hyphal length per AM fungal colonized root length was calculated by dividing the total hyphal length for each compartment by the corresponding AM fungal colonized root length.

**Methods S3**

*RT-qPCR validation of* MtZIP1*,* MtZIP2, *MtZIP14 and* MtNAS1

The primer pairs were tested on a mixed sample of shoot and root cDNA of *Medicago truncatula* in three replicates. The specificity of amplification was successfully assessed by Sanger sequencing of the PCR amplicons using an ABI 3730 XL Applied Biosystem (Eurofins, Germany) and by melting curve analysis at the end of each qPCR run. The concentration ranges in which the relationship between the relative fluorescence and the logarithm of the concentration is linear and the precision of quantification (standard curves), as reflected in the coefficient of correlation (R^2^), were determined using three independent 10-fold serial dilutions of the cDNA. The accuracy of quantification was determined by the efficiency (E) of each qPCR amplification, using the equation E = [10−1/S − 1] × 100, where S is the slope of the standard curve.

**Methods S4**

*RT-qPCR primer design for the Rhizophagus irregularis genes* RiZnT1 *and* RiZRT1

The sequences of the RiZnT1 and RiZRT1 genes were downloaded from GenBank (NCBI; <https://www.ncbi.nlm.nih.gov/genbank/>) and specific primer pairs were designed using the Primer-BLAST online tool (<https://www.ncbi.nlm.nih.gov/tools/primer-blast/>).

*Validation of the new RT-qPCR assays for the Rhizophagus irregularis genes* RiZnT1 *and* RiZRT1

PCR amplifications were performed from each cDNA sample using the primer pairs reported in Table S6 (three technical replicates per each PCR amplification), targeting the selected genes. PCR amplicons were generated in volumes of 20 µL with 0.5 U of HotStarTaq DNA Polymerase (Qiagen, Venlo, Netherlands), 10 µM of each primer, 0.2 mM of each dNTP, 1 mM of MgCl2 and 1x reaction buffer, using a S1000 Thermal Cycler TM (BIORAD, Hercules, CA, USA). The thermal cycler was programmed as follows: 95 °C for 2 min, 30 cycles at 95 °C for 45 s, 60 °C for 45 s, 70 °C for 1 min and a final extension step at 72 °C for 10 min. Reaction yields and fragment lengths were estimated using a 1% agarose gel electrophoresis, containing Sybr Safe (Invitrogen, Carlsbad, CA).

The RT-qPCRs for gene expression analysis were run using each cDNA sample (three technical replicates) with a final reaction volume of 20 μL, 10 μL of SYBR Green Supermix (Biorad), 5 μL of 100-fold diluted cDNA, and 0.4 μM final concentrations of the gene-specific PCR primers on a CFX Connect Real-Time System thermal cycler (Biorad, Hercules, California). The qPCR conditions were 95°C for 3’, followed by 40 cycles of 95° C for 5’, and 60° C for 30’’. A dissociation curve of each reaction was performed (65° C to 95° C, 0.5° C increment every 5’’) to check that PCR amplified only one product. Thus, the specificity of amplification of the primer pair was assessed by Sanger sequencing of the PCR amplicons (Eurofins, Germany) and by the melting curve analysis at the end of each qPCR run. The coefficients of correlation (R^2^) and the efficiency (E) of each qPCR amplification were determined using three independent 5-fold serial dilutions.

**Methods S5**

*RNA isolation and real-time RT-PCR*

Possible traces of DNA in the RNA extracts were removed by a DNase treatment (Promega, USA). The purity of the RNA extracts was verified using NanoDrop 2000 (Thermo Scientific, Worchester, MA, USA). Extracted RNA was loaded in a 1% agarose gel electrophoresis with Sybr Safe (Invitrogen, Carlsbad, CA), to check integrity and approximate concentration.

The RT-qPCRs for gene expression analysis were run in a volume of 20 μL, 10 μL of SYBR Green Supermix (Biorad), 5 μL of diluted cDNA (50-fold dilution for plant genes and 10-fold dilution for fungal genes), and 0.4 μM final concentrations of the gene-specific PCR primers (Table S6) on a CFX Connect Real-Time System thermal cycler (Biorad, Hercules, California). The qPCR conditions were 95° C for 3 min, followed by 40 cycles of 95° C for 5 min, and 60° C for 30 s. A dissociation curve of each reaction was performed (65° C to 95° C, 0.5° C increment every 5 s) to check that the PCR amplified only one product.

**References**

Catoira, R., Galera, C., de Billy, F., Penmetsa, R. V., Journet, E. P., Maillet, F., Rosenberg, C. (2000) Four genes of *Medicago truncatula* controlling components of a Nod factor transduction pathway. The Plant Cell. 12: 1647-1665.

Cranenbrouck, S., Voets, L., Bivort, C., Renard, L., Strullu, D.G., and Declerck, S. (2005) Methodologies for in vitro cultivation of arbuscular mycorrhizal fungi with root organs. In *In Vitro* Culture of Mycorrhizas. Declerck, S., Fotin, A., Strullu, D.G. (eds). Heidelberg, Germany: Springer, pp. 341-375.

Morandi, D., Prado, E., Sagan, M., and Duc, G. (2005) Characterisation of new symbiotic *Medicago truncatula* (Gaertn.) mutants, and phenotypic or genotypic complementary information on previously described mutants. Mycorrhiza 15: 283-289.

Sagan M, de Larembergue H, and Morandi D (1998) Genetic analysis of symbiosis mutants in *Medicago truncatula*. In Biological nitrogen fixation for the 21^st^ century. Elmerich, C., Kondorosi, A., and Newton, W.E. (eds). Kluwer, Dordrecht, pp 317-318.

Sagan, M., Morandi, D., Tarenghi, E., and Duc, G. (1995) Selection of nodulation and mycorrhizal mutants in the model plant *Medicago truncatula* (Gaertn.) after γ-ray mutagenesis. Plant Sci. 111: 63-71.
